# Optimal exercise prescription for balance after stroke: a Bayesian dose–response network meta-analysis

**DOI:** 10.3389/fneur.2026.1804224

**Published:** 2026-04-13

**Authors:** Chunbai Xue Yang, Yang Xu, Zhongwei Zhao, Yingwei Zhu

**Affiliations:** 1 School of Physical Education, Bohai University, Jinzhou, Liaoning, China; 2 School of Physical Education, Shenyang Normal University, Shenyang, Liaoning, China

**Keywords:** aerobic exercise, balance, Berg Balance Scale, exercise dose, exercise therapy, resistance training, stroke, stroke rehabilitation

## Abstract

**Background:**

Post-stroke balance impairment is common and clinically consequential, contributing to increased fall risk, reduced functional independence, and long-term disability. Exercise is widely prescribed to improve balance after stroke, yet the dose required for meaningful benefit—and whether higher doses yield additional gains—remains uncertain due to heterogeneous exercise prescriptions across trials.

**Objective:**

To quantify the dose–response relationship between exercise dose (standardized as METs-min/week) and balance improvement after stroke, and to estimate the minimum effective dose and the dose range associated with maximal benefit using Bayesian model-based dose–response meta-analysis.

**Methods:**

We searched PubMed/MEDLINE, Embase, Web of Science Core Collection, Scopus, CENTRAL, and major Chinese databases, from inception to December 31, 2025, and conducted backward citation tracking. We included randomized controlled trials enrolling stroke survivors. The primary outcome was the Berg Balance Scale (BBS). We used Bayesian random-effects network meta-analysis to compare exercise modalities and Bayesian model-based methods to estimate dose–response relationships. Risk of bias was assessed using RoB 2, and certainty of evidence was evaluated with CINeMA.

**Results:**

We included 42 randomized controlled trials. Overall, the dose–response relationship was non-linear: the predicted effect suggested an increase with dose up to an apparent peak region around ~1,200 MET-min/week, followed by attenuation/decline at higher doses, with greater uncertainty where high-dose data were sparse. In the ascending portion of the curve, the model-implied average local change was approximately +0.12 Hedges’ g per 100 MET-min/week, whereas beyond the peak region it was approximately −0.05 per 100 MET-min/week. The minimum dose associated with a credible improvement was ~270 MET-min/week, within the observed dose range of included trials. Across modalities, resistance training showed the largest pooled benefit versus control, while aerobic, resistance, and water-based exercise exhibited non-linear patterns with modality-specific peak regions; Chinese exercise and balance training showed positive associations within the evidence-supported dose range. Estimates for HIIT were small and generally imprecise, with credible intervals frequently including no effect.

**Conclusion:**

Exercise significantly improves balance in patients with stroke, with ≥270 METs-min/week representing the credible minimum effective dose. Based on our dose-informed recommendations, clinical programs may prioritize moderate-intensity training performed 3–5 times per week, with resistance training (RT) and water-based exercise (WBE) most likely to yield larger gains (predicted peak doses: RT ~ 666 METs-min/week; WBE ~ 1,616 METs-min/week), while aerobic exercise (AE) is also effective at moderate doses (predicted peak ~554 METs-min/week). In addition, Chinese exercise (CE) and balance training (BT) show a stable positive dose–effect association within the evidence-supported range. By contrast, HIIT shows small and uncertain effects and is therefore not recommended as a routine first-line option at present.

**Systematic review registration:**

PROSPERO, CRD420261297953.

## Introduction

Stroke is among the leading causes of death and long-term disability worldwide. Many survivors are left with varying degrees of motor and functional impairments that substantially compromise independent living and social participation (1). Among post-stroke functional deficits, impaired balance is particularly common, manifesting as reduced postural control, limited ability to shift the center of mass, and insufficient gait stability—thereby increasing fall risk and prolonging rehabilitation (2). Falls may not only result in fractures, soft-tissue injury, and rehospitalization, but can also trigger fear of movement, avoidance behaviors, and diminished quality of life, ultimately reinforcing a vicious cycle of “reduced activity—deconditioning—recurrent falls (3).” Consequently, identifying feasible, implementable exercise/training strategies that effectively improve balance function after stroke remains a long-standing priority in stroke rehabilitation (4).

Physical activity and exercise are central to stroke rehabilitation. According to classical definitions, physical activity encompasses any bodily movement produced by skeletal muscle contraction that results in energy expenditure above resting levels, whereas exercise is planned, structured, and repetitive, with the goal of improving or maintaining physical fitness and function (5, 6). Stroke rehabilitation guidelines and scientific statements generally recommend that, following appropriate safety assessment, stroke survivors be supported to progressively resume and sustain regular exercise, integrating aerobic, strengthening, and functional training to improve fitness and functional outcomes (7–9). From a public-health perspective, the World Health Organization (WHO) recommends that adults achieve a specified weekly volume of moderate-to-vigorous physical activity and emphasizes that people living with chronic conditions and disability should likewise be supported to engage in physical activity under individualized considerations (10, 11). However, translating these broad “recommended amounts” into a clinically actionable “prescription dose” specifically for post-stroke balance rehabilitation remains constrained by limited evidence and suboptimal operationalizability in routine practice (12).

Rehabilitation interventions targeting post-stroke balance are diverse, including aerobic training, resistance training, high-intensity interval training, balance-specific training, and modalities with distinct cultural and contextual features (13–15). Systematic reviews and meta-analyses indicate that targeted exercise therapy can, overall, improve balance in people with chronic stroke, although effects may differ across training components and protocols (16, 17). From an evidence-synthesis standpoint, multiple reviews have summarized exercise-based interventions for post-stroke balance; nevertheless, conclusions are often limited by between-trial heterogeneity, complex intervention combinations, and inconsistent outcome measures and scales (18). Evidence for fall prevention in stroke populations similarly suggests that exercise may be beneficial; however, overall certainty remains limited, and robust, comparable dose information across exercise programs is frequently lacking (19, 20).

Critically, two evidence gaps continue to impede clinical prescribing. First, the comparative effects of different exercise modalities on balance remain insufficiently resolved. Conventional pairwise meta-analyses typically contrast “one exercise type versus control,” limiting simultaneous comparisons and precluding coherent ranking across competing options; by contrast, network meta-analysis synthesizes direct and indirect evidence within a common-comparator framework, enabling multi-intervention comparison (21). Second—and more directly aligned with prescription—the dose–response relationship has not been reliably delineated. Prior reviews often handle dose (an inherently continuous variable) via coarse categorization or rely on linear assumptions, potentially obscuring non-linear patterns. Low doses may be insufficient to elicit functional change, whereas excessive doses may yield diminishing returns due to fatigue, reduced adherence, or safety concerns. In stroke rehabilitation, “more is better” is not axiomatic; therefore, defining a minimum effective dose and a potential optimal dose range is essential for resource optimization and clinical feasibility (22).

Recent advances in Bayesian model-based network meta-analysis (MBNMA) offer a pragmatic means to address both multi-intervention comparison and dose–response modeling. By embedding dose–response functions within the network, MBNMA can estimate relative effects across dose levels, generating evidence more directly aligned with prescription-oriented decision-making (23). Network evidence synthesis must also interrogate key assumptions—particularly consistency and potential inconsistency—to ensure the statistical and clinical comparability and credibility of direct and indirect evidence (24).

Accordingly, this study will systematically identify randomized controlled trials and apply a Bayesian model-based dose–response network meta-analysis to quantify the effects of different exercise modalities, across dose levels, on balance after stroke. We will compare and rank intervention effectiveness and identify clinically meaningful dose ranges on interpretable dose scales, thereby providing more actionable evidence to inform the development and optimization of exercise prescriptions for post-stroke balance training (25).

## Methods

### Design

This systematic review and meta-analysis was conducted in accordance with the Preferred Reporting Items for Systematic Reviews and Meta-Analyses (PRISMA) guideline (26). The review protocol was registered with PROSPERO (registration number: CRD420261297953).

### Eligibility criteria

We conducted a systematic review and a dose–response meta-analysis of randomized controlled trials (RCTs) to evaluate the association between exercise-intervention dose and improvements in balance function among people with stroke. Eligibility criteria were: (i) an RCT design; (ii) participants with stroke; (iii) a clearly defined exercise or training program, including aerobic exercise, resistance exercise, balance training, aquatic-based training, or high-intensity interval training; (iv) clearly reported and quantifiable dose information, including training frequency, session duration, and intervention period (or sufficient data to derive weekly training volume); (v) a strict no-exercise control, defined as receiving no structured exercise or training intervention (usual care permitted); and (vi) balance-related outcomes reported in a form allowing effect-size calculation and inclusion in the dose–response analysis. No restrictions were imposed on publication year.

To enhance the comparability and interpretability of dose–response estimates, we excluded studies using active comparators or dose-matched exercise controls. We also excluded non-randomized designs and crossover trials lacking usable parallel-group control data; studies assessing only the acute effects of a single exercise bout; trials in which exercise was combined with other intensive co-interventions (e.g., pharmacotherapy, devices, or high-intensity cognitive training) such that the independent effect of exercise could not be isolated; and studies with completely missing key dose components (training frequency, session duration, or intervention period) for which the authors could not provide additional information.

### Information sources

We systematically searched the following electronic databases from inception to December 31, 2025: PubMed, Embase, Web of Science Core Collection, Scopus, and the Cochrane Central Register of Controlled Trials (CENTRAL). We also searched the Chinese databases CNKI, Wanfang, and VIP. In addition to structured database searches, we conducted backward citation tracking by screening the reference lists of included studies and relevant systematic reviews to identify any eligible studies that may have been missed.

### Search strategy

The search strategy was structured around three core concepts: stroke (e.g., stroke, cerebrovascular accident), balance function or balance assessment (e.g., balance, postural control, Berg Balance Scale [BBS]), and exercise/rehabilitation training interventions (e.g., exercise, rehabilitation, aerobic training, resistance training, balance training, high-intensity interval training, traditional Chinese exercise, aquatic or water-based exercise), combined with an RCT study-design filter (e.g., randomized controlled trial, RCT). For each database, we used both controlled vocabulary (e.g., MeSH and Emtree terms) and free-text keywords, adapting search fields and syntax to database-specific indexing and platform requirements. The complete search strategies and search dates are reported in the Supplementary File 1.

### Selection process

After deduplication, two authors (CBXY and YX) independently screened records by titles/abstracts and subsequently assessed potentially eligible articles in full text. Reference lists of included studies were also screened (backward citation tracking) to identify additional eligible trials. At each stage, studies were evaluated against prespecified inclusion and exclusion criteria, and reasons for full-text exclusion were recorded to generate the PRISMA flow diagram. Disagreements were resolved by discussion; if consensus could not be reached, a third reviewer adjudicated.

### Data extraction

Two authors (CBXY and YX) independently extracted data from studies meeting the eligibility criteria. Discrepancies were resolved by consensus and, when necessary, adjudicated by a third author (YWZ). From each included study, we extracted participant characteristics (e.g., age, sex, baseline balance level), descriptions of the intervention and control conditions (exercise modality, frequency, session duration, intervention duration in weeks, and intensity or sufficient information to derive/standardize intensity), the balance outcome measure(s), and data required to compute effect sizes (group means, standard deviations, and sample sizes for intervention and control groups, or equivalent convertible statistics). When published reports lacked the minimum information required for dose–response analysis (e.g., frequency/duration/intensity could not be determined, or outcomes were presented only graphically without extractable numeric values), we contacted corresponding authors to request additional data; if key data could not be obtained, the study was excluded from the corresponding analysis.

### Data analysis

We classified interventions using a three-level coding scheme. At Level 1, interventions were coded as “exercise” or “control.” At Level 2, exercise interventions were coded by primary modality: “resistance training,” “aerobic training, or “mixed exercise,” with “control” retained as the reference category. At Level 3, interventions were coded at the intersection of modality and dose. Dose was defined as energy expenditure expressed in metabolic equivalents of task (METs) and operationalized as MET-min/week, calculated as the product of session duration, training frequency, and exercise intensity. To improve network connectivity—a prerequisite for network meta-analysis—we mapped each intervention’s estimated MET-min/week to the nearest prespecified dose category: 500, 750, 1,000, 2000, or 2,500 MET-min/week.

### Risk of bias assessment

Two reviewers (CBXY and YX) independently assessed the risk of bias of the included studies using the revised Cochrane Risk of Bias tool for randomized trials (RoB 2), covering five domains: (i) bias arising from the randomization process; (ii) bias due to deviations from intended interventions; (iii) bias due to missing outcome data; (iv) bias in measurement of the outcome; and (v) bias in selection of the reported result. Each domain was judged as “low risk,” “some concerns,” or “high risk,” and an overall judgment for each study was derived according to the RoB 2 signaling questions and algorithm. Discrepancies were resolved through discussion and, when necessary, adjudicated by a third reviewer (YWZ).

### Certainty of evidence

We used the CINeMA (Confidence in Network Meta-Analysis) framework to rate the certainty of evidence for each network effect estimate. CINeMA assesses certainty across six domains: within-study bias, reporting bias, indirectness, imprecision, heterogeneity, and incoherence. Within-study bias was primarily informed by the RoB 2 assessments; the remaining domains were judged by integrating network meta-analysis outputs with the percentage contribution of evidence within the network. Because our network was anchored to a single common comparator (no structured exercise, usual care permitted) and contained no closed loops, incoherence (inconsistency) could not be assessed; therefore, CINeMA ratings were based on the remaining assessable domains, and incoherence was transparently reported as “not assessable.” The overall certainty of evidence for each comparison was categorized as high, moderate, low, or very low.

### Statistical analysis

All analyses were conducted in R (version 4.0.3), and the primary manuscript results were derived from this prespecified analytic pipeline. Although all included studies used the Berg Balance Scale (BBS), making mean differences (MDs) on the original scale directly clinically interpretable, the prespecified primary analyses were conducted on the standardized mean difference scale (Hedges’ g) to maintain a common effect-size framework across the Bayesian network meta-analysis (NMA) and model-based dose–response network meta-analysis (MBNMA), while applying a small-sample correction (27). Effect sizes and corresponding sampling variances were calculated using the metafor package. A supplementary sensitivity analysis using MDs on the original BBS scale was additionally performed to assess clinical interpretability and robustness to the choice of effect-size metric (Supplementary File 8).

We first performed a Bayesian random-effects NMA to synthesize relative effects across exercise modalities, reporting posterior means with 95% credible intervals (CrIs) (28). Using the control group (CG) as the reference, pooled network effects were presented in forest plots and pairwise relative effects in a league table. Ranking results, summarized using posterior rank probabilities and SUCRA values (23), were treated as descriptive because the network was star-shaped, anchored on CG, and contained no closed loops.

We then conducted a Bayesian random-effects MBNMA using the MBNMAdose package to characterize the dose–response relationship between exercise dose and balance outcomes; dose–response curves were visualized using ggplot2 (29, 30). In the primary MBNMA, dose was modeled continuously on the MET-min/week scale. Discretized dose nodes were used only for split-NMA connectivity checks, visualization, and exploration of candidate functional forms (Supplementary Files 2, 3), and not as the primary exposure scale for interpretation.

Connectivity and model fit were examined by comparing consistency and unrelated mean effects (UME) models using the deviance information criterion (DIC), residual deviance, and the effective number of parameters (pD) (Supplementary File 2). Because the network contained no closed loops, formal inconsistency assessment methods based on loop structures, including node-splitting, were not applicable.

To inform functional-form selection, we performed a split-NMA treating each modality × dose group as an independent node and compared candidate functions (e.g., Emax, exponential, restricted cubic splines, and non-parametric monotonic functions) using model-fit indices and diagnostic plots (Supplementary File 3). Model-predicted effects were summarized on the continuous dose scale. Predictions at selected reference doses spanning the observed range are provided in the Supplementary materials as communication anchors rather than clinical thresholds. Potential small-study effects were assessed by visual inspection of a dose-adjusted contour-enhanced funnel plot of meta-regression residuals.

To assess robustness to risk of bias, we conducted a prespecified sensitivity analysis excluding trials rated as overall high risk of bias under RoB 2. Because domain-specific exclusions could substantially fragment sparse modality networks, sensitivity analyses were prespecified at the overall RoB level. We refit the modality-level pooled comparison models and the overall dose–response model using the reduced dataset and compared these findings with the primary analyses (Supplementary File 8).

Additional computational verification and convergence diagnostics are provided in Supplementary File 9. The primary manuscript analyses were conducted in R, whereas the Python-based implementation documented in Supplementary File 9 was used only as a supplementary transparency, verification, and diagnostic workflow. Convergence was evaluated using R-hat, effective sample size (ESS), and representative trace and posterior density plots (Supplementary Table 9 and Supplementary Figure 11).

## Results

### Study selection

**Figure 1:**
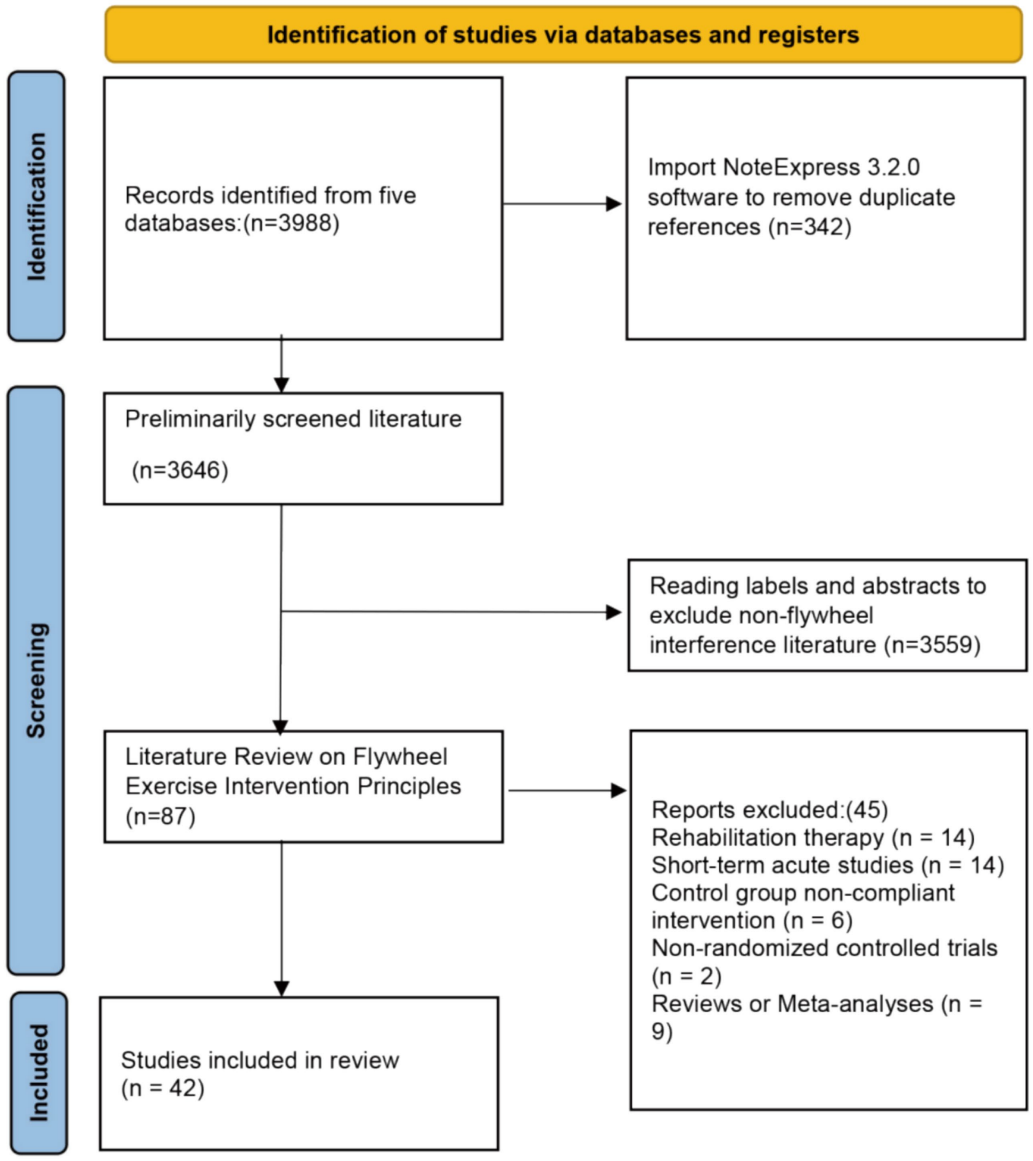
PRISMA flow diagram.

The study selection process is presented in the PRISMA flow diagram (Figure 1). Database searches across eight sources identified 3,988 records. Reference lists of included studies (and relevant systematic reviews, where applicable) were screened, yielding five additional studies. After removal of 3,699 duplicates, 289 records were screened by title and abstract. Full texts of 87 articles were retrieved and assessed for eligibility, and 42 studies enrolling participants with stroke were included in the quantitative synthesis to evaluate the effects of different exercise interventions on post-stroke balance function.

### Study characteristics

The included studies investigating exercise interventions in stroke patients all employed experimental and control groups and reported participants’ age, sample size, and sex distribution. Participants comprised predominantly middle-aged and older adults, with sample sizes ranging from 7 to 54 in the experimental groups and 5 to 56 in the control groups, and a higher proportion of males than females. Intervention modalities included aerobic exercise, resistance training, balance training, high-intensity interval training (HIIT), aquatic exercise (water-based exercise, WBE), and traditional Chinese exercise (Chinese exercise, CE). Session durations varied by intervention type, ranging from 15 to 60 min for most interventions, with one study reporting up to 120 min per session. Training frequency ranged from 3 to 10 sessions per week, and intervention periods spanned 2 to 19 weeks. Reported weekly exercise doses ranged from 262.5 to 2,520 MET-min/week, with some studies not providing dose information; after dose mapping, weekly doses were primarily concentrated at 500, 750, 1,000, 1,500, and 2000 MET-min/week, corresponding to daily doses of 37.5–360 MET-min/day. All studies employed the Berg Balance Scale (BBS) as the primary outcome measure to assess balance and postural control in stroke patients. Detailed information on study characteristics, intervention protocols, and exercise parameters is provided in Online Supplementary File 4.

### Risk of bias assessment

**Figure 2:**
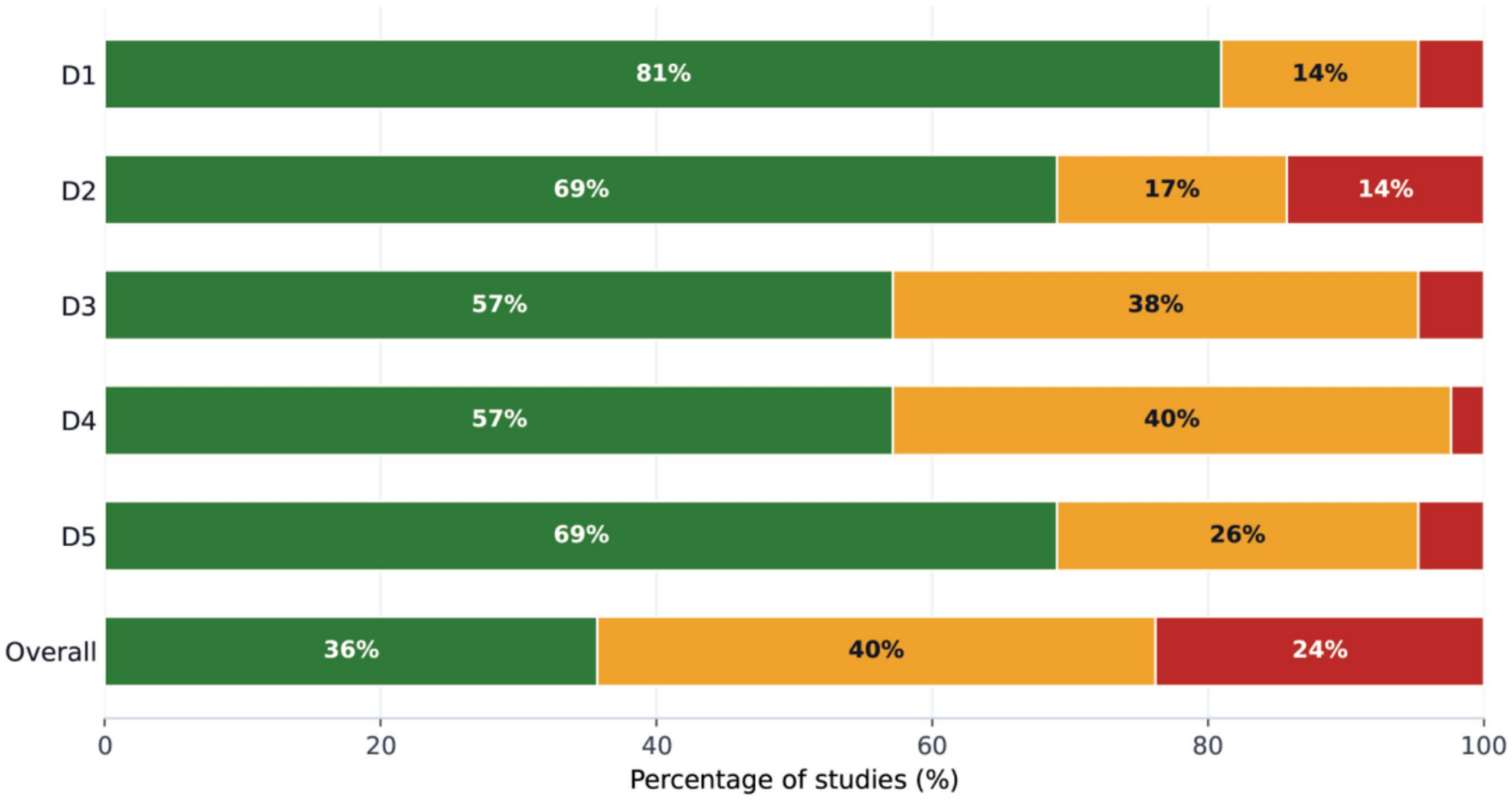
Distribution of ROB 2 domain-level and overall risk-of-bias judgements. This figure shows the proportion of included studies across the ROB 2 risk-of-bias domains (D1–D5) and the overall judgement. The *x*-axis represents the percentage of studies (%). Stacked bars are segmented by judgement category: low risk, some concerns, and high risk; values within bars indicate the corresponding percentages. D1–D5 denote the following domains: D1 bias arising from the randomization process, D2 bias due to deviations from intended interventions, D3 bias due to missing outcome data, D4 bias in measurement of the outcome, and D5 bias in selection of the reported result.

Figure 2 summarizes RoB 2 judgements across domains (D1–D5) and overall risk of bias. Overall, 36% of studies were judged as low risk, 40% as some concerns, and 24% as high risk. At the domain level, the proportion rated as high risk was 5% for the randomization process (D1), 14% for deviations from intended interventions (D2), 5% for missing outcome data (D3), 3% for measurement of the outcome (D4), and 5% for selection of the reported result (D5). The distribution of judgements differed across domains, with comparatively higher proportions of some concerns in missing outcome data (D3; 38%) and measurement of the outcome (D4; 40%). Study-level domain judgements and overall ratings are reported in Supplementary File 5.

### Certainty of evidence

**Figure 3:**
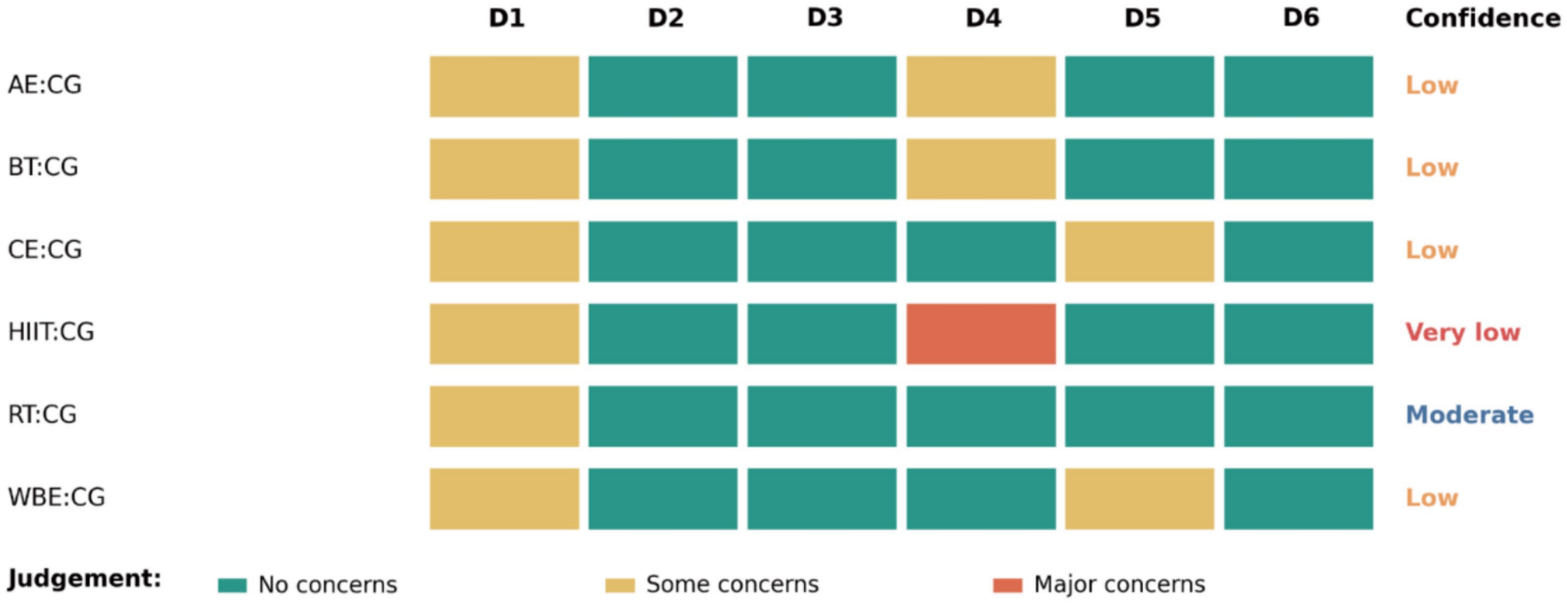
CINeMA six-domain judgements and overall confidence for comparisons versus control. Heatmap summarizing the CINeMA assessment for each intervention compared with the control group (CG). Rows represent treatment–control comparisons (AE, BT, CE, HIIT, RT, and WBE vs. CG), and columns D1–D6 correspond to CINeMA domains: D1 within-study bias, D2 reporting bias, D3 indirectness, D4 imprecision, D5 heterogeneity, and D6 incoherence. Cell colors indicate domain-level judgements (no concerns, some concerns, major concerns). The rightmost column reports the overall confidence rating for each comparison, derived from the pattern and severity of domain-level concerns.

**Figure 4:**
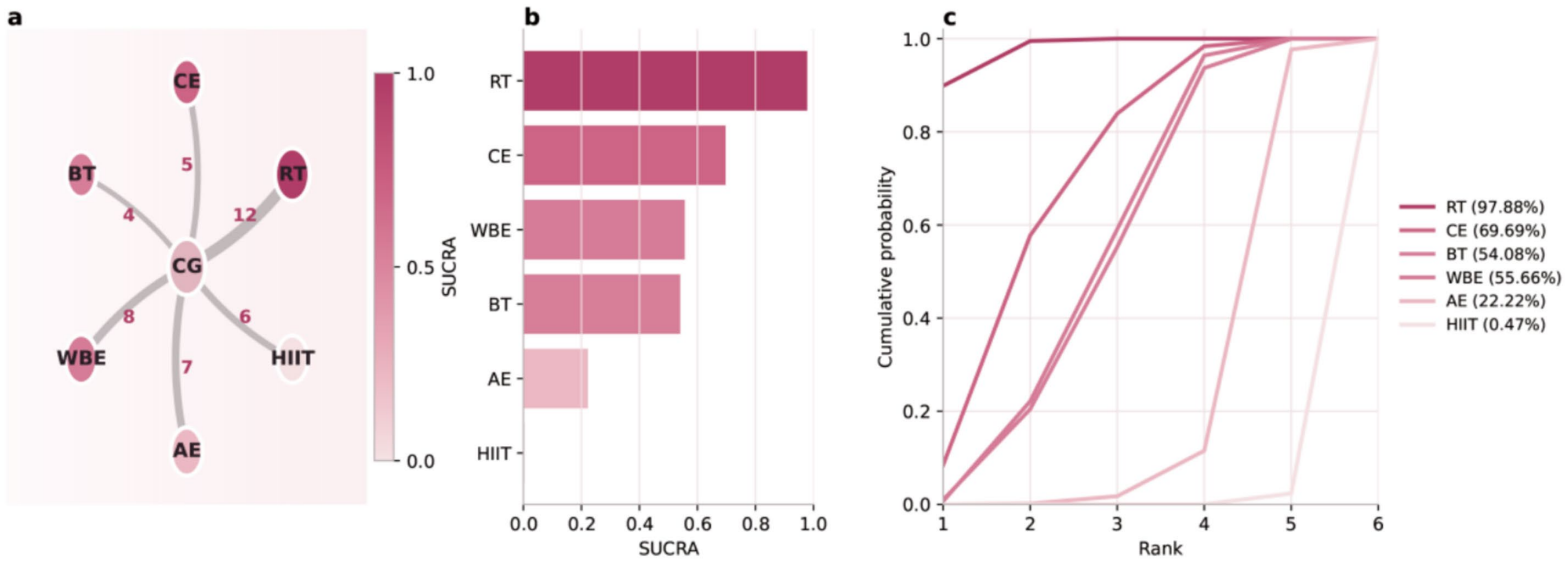
Evidence network, SUCRA ranking, and rankograms across exercise modalities. **(a)** Evidence network plot for the network meta-analysis: nodes represent interventions (RT, CE, BT, WBE, AE, HIIT, and CG), and edges indicate the presence of direct comparisons. Numbers adjacent to the edges denote the number of trials for each comparison (color intensity reflects relative ranking/estimated efficacy). **(b)** Bar chart of SUCRA values for each intervention; higher SUCRA indicates a higher overall ranking. **(c)** Cumulative ranking probability curves (rankograms); curves that lie further to the left and rise more rapidly indicate a higher probability of achieving better ranks. SUCRA percentages are shown in parentheses in the legend.

Figure 3 presents CINeMA judgements for comparisons against the control group (CG). Overall confidence was moderate for RT vs. CG, low for AE, BT, CE, and WBE vs. CG, and very low for HIIT vs. CG. Domain-level concerns were concentrated in within-study bias (D1) and imprecision (D4), whereas indirectness (D3) and incoherence (D6) were generally judged as no concerns. Full domain judgements, confidence ratings, and downgrading rationale are provided in Supplementary File 5 and Figure 4.

### Network meta-analysis

Figure 3a depicts the evidence network for balance outcomes across exercise modalities in people with stroke. Nodes represent interventions (RT, CE, WBE, BT, AE, HIIT) and the control condition (CG); edges indicate available direct head-to-head comparisons, and line thickness is proportional to the number of studies contributing direct evidence.

**Figure 5:**
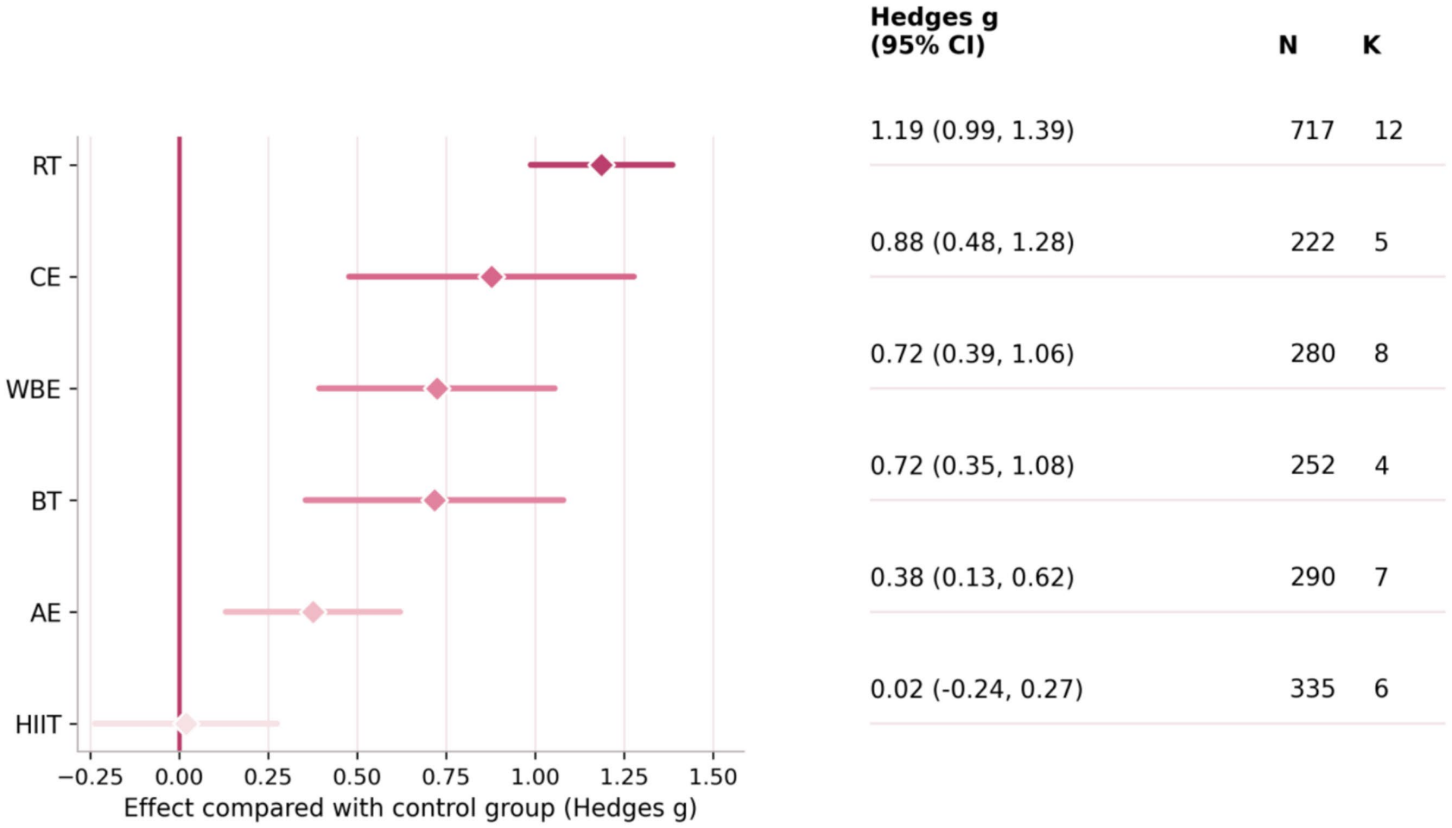
Forest plot of pooled effects of exercise interventions versus control. Forest plot of pooled effects of each exercise intervention compared with the control group (CG). Points indicate pooled standardized mean differences (Hedges’ *g*) and horizontal lines represent 95% confidence intervals (CIs); the vertical line denotes the null effect (*g* = 0). The total sample size (*N*) and number of included studies (*K*) for each intervention are shown on the right. Overall, larger pooled improvements were observed for RT (*g* = 1.19), CE (*g* = 0.88), WBE (*g* = 0.72), and BT (*g* = 0.72), a moderate effect for AE (*g* = 0.38), and an estimate close to null for HIIT (*g* = 0.02). Effects should be interpreted in the context of their CIs.

After integrating direct and indirect evidence within a Bayesian random-effects network model, the forest plot in Figure 5 summarizes pooled effects versus control (Hedges’ g with 95% credible intervals [CrIs]), along with sample sizes and numbers of studies. RT showed the largest improvement in balance (*g* = 1.19, 95% CrI 0.99–1.39; *N* = 717; *K* = 12), followed by CE (*g* = 0.88, 0.48–1.28; *N* = 222; *K* = 5), WBE (*g* = 0.72, 0.39–1.06; *N* = 280; *K* = 8), and BT (*g* = 0.72, 0.35–1.08; *N* = 252; *K* = 4). AE was also associated with improvement (*g* = 0.38, 0.13–0.62; *N* = 290; *K* = 7), whereas HIIT was close to null and not credibly different from no effect (*g* = 0.02, −0.24 to 0.27; *N* = 335; *K* = 6). Table 1 presents the full set of pairwise relative effect estimates from the Bayesian network meta-analysis. Figures 3b,c show posterior ranking probabilities and cumulative ranking curves: RT had the highest probability of being ranked best (SUCRA = 97.88%), followed by CE (69.69%), BT (64.08%), WBE (55.66%), and AE (22.22%), with HIIT ranking lowest (0.47%). Overall, within the current evidence network, RT was most likely to yield the greatest pooled benefit for balance after stroke; these findings should be interpreted alongside the dose–response results. Posterior rank probabilities are presented as descriptive summaries in Supplementary File 7 and should be interpreted cautiously given the star-shaped network structure without closed loops.”

**Table 1:** Pairwise and network meta-analysis results.

	**Pairwise meta-analysis**						
Network meta-analysis	**CE**	**/**	**/**	**/**	**/**	**/**	**0.90 (0.51, 1.29)**
	**0.49 (0.03, 0.96)**	**AE**	**/**	**/**	**/**	**/**	**0.41 (0.16, 0.66)**
	0.22 (−0.29, 0.73)	−0.27 (−0.68, 0.14)	**WBE**	**/**	**/**	**/**	**0.68 (0.35, 1.01)**
	**0.87 (0.43, 1.32)**	**0.38 (0.05, 0.71)**	**0.65 (0.26, 1.04)**	**HIIT**	**/**	**/**	0.03 (−0.19, 0.25)
	0.18 (−0.35, 0.71)	−0.32 (−0.75, 0.12)	−0.04 (−0.53, 0.44)	**−0.69 (−1.11, −0.28)**	**BT**	**/**	**0.72 (0.37, 1.08)**
	−0.30 (−0.74, 0.13)	**−0.80 (−1.12, −0.48)**	**−0.53 (−0.91, −0.14)**	**−1.18 (−1.47, −0.88)**	**−0.48 (−0.89, −0.08)**	**RT**	**1.21 (1.01, 1.40)**
	**0.90 (0.51, 1.29)**	**0.41 (0.16, 0.66)**	**0.68 (0.35, 1.01)**	0.03 (−0.19, 0.25)	**0.72 (0.37, 1.08)**	**1.21 (1.01, 1.40)**	**CG**

The diagonal lists the intervention categories (CE, AE, WBE, HIIT, BT, RT, and CG). The upper-right triangle presents effect estimates and 95% confidence intervals (CIs) from pairwise meta-analyses based on direct evidence; “/” indicates that no direct comparison was available. The lower-left triangle presents effect estimates and 95% CIs from the network meta-analysis, synthesizing both direct and indirect evidence. CG denotes the control group. The direction (positive vs negative) of the effect estimate follows the prespecified effect definition in this study. Bold values indicate statistically significant effects (95% CI does not include 0).

### Dose–response relationship and minimum clinically important difference

**Figure 6:**
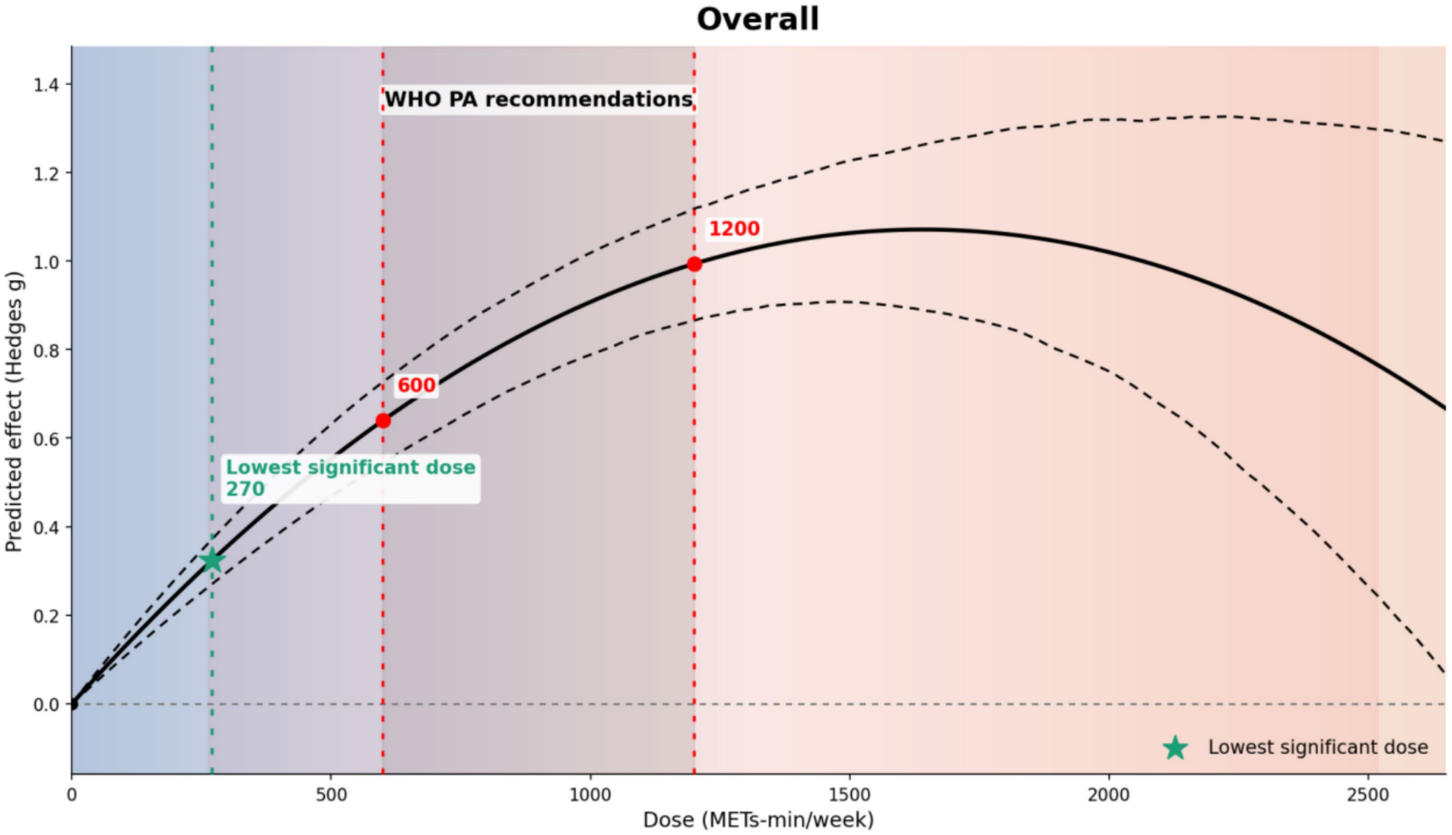
Overall dose–response relationship between exercise dose. Overall dose–response relationship. The *x*-axis shows exercise dose (MET·min/week) and the *y*-axis shows the model-predicted effect size (Hedges’ *g*). The solid line represents the estimated dose–response curve and dashed lines indicate the 95% credible interval; the horizontal grey dashed line denotes the null effect (*g* = 0). The green star marks the lowest statistically significant dose (approximately 270 MET·min/week). Red vertical dashed lines indicate the WHO-referenced physical activity range (approximately 600–1,200 MET·min/week), and red points show the corresponding model-predicted effects at these dose nodes. Shaded background regions are provided to facilitate visual interpretation of trends across dose ranges. Predicted effects at higher doses should be interpreted cautiously where data are sparse and uncertainty widens.

Figure 6 shows the dose–response relationship between exercise dose and balance improvement. The predicted effect increased with dose up to approximately 1,200 METs-min/week (average change ≈ 0.12 in Hedges’ g per 100 METs-min/week within this range). Beyond 1,200 METs-min/week, higher doses were not associated with further gains; instead, the predicted effect declined (average change ≈ − 0.05 per 100 METs-min/week), and no plateau was evident within the predicted range. The minimum dose at which the predicted effect became credibly greater than zero (i.e., the 95% CrI excluded zero) was 270 METs-min/week. At 600 METs-min/week (WHO lower bound), the predicted effect was moderate (*g* = 0.72, 95% CrI [0.69, 0.74]; posterior SD = 0.01), whereas a larger effect was observed at 1200 METs-min/week (WHO upper bound) (*g* = 1.44, 95% CrI [1.38, 1.49]; posterior SD = 0.03). When the dose increased to 1800 METs-min/week (approximately twice the WHO lower bound), the predicted effect decreased (*g* = 1.11, 95% CrI [1.02, 1.19]).

**Figure 7:**
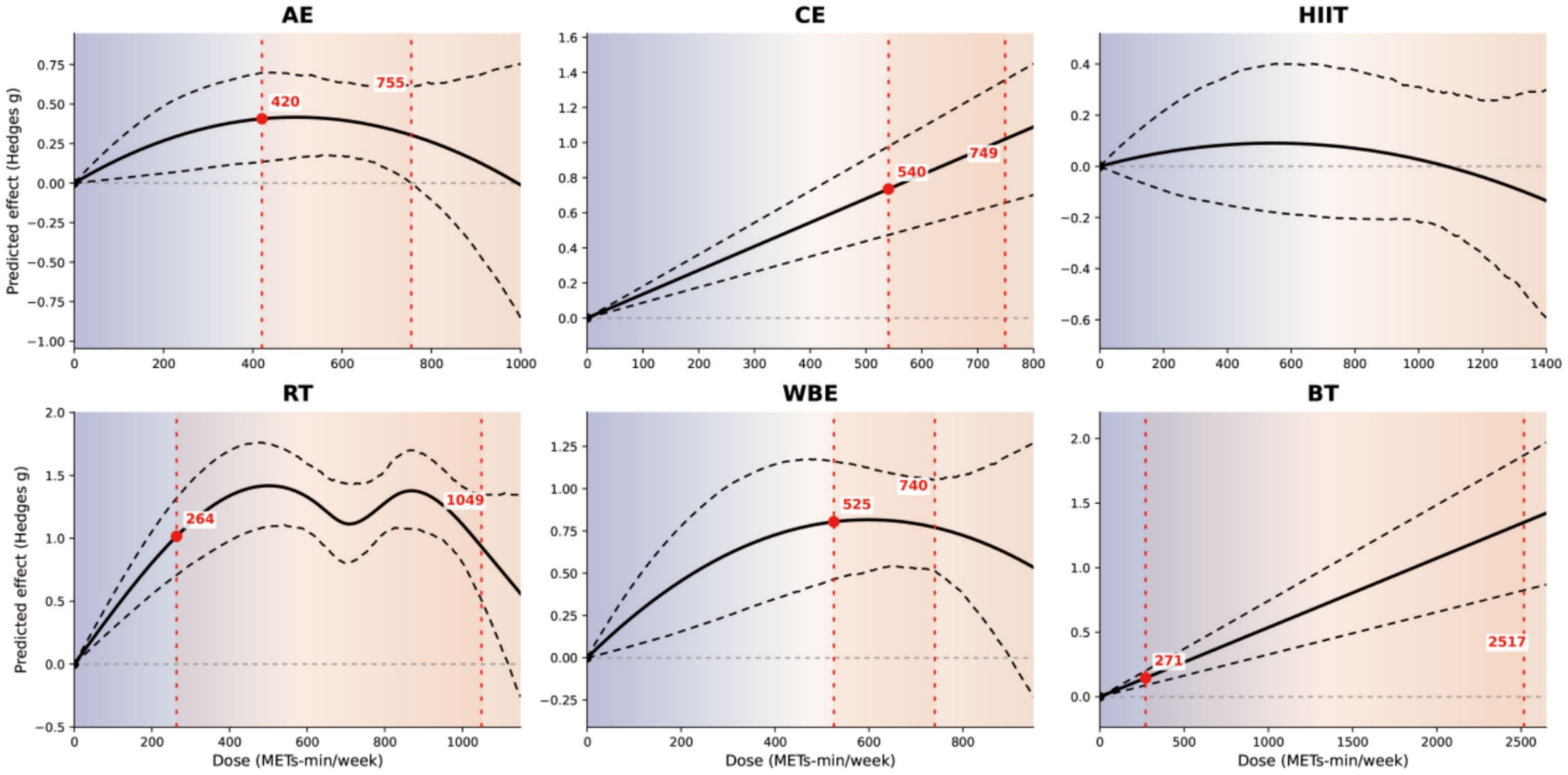
Modality-specific dose–response relationships. Dose–response curves are shown separately for AE, CE, HIIT, RT, WBE, and BT. The *x*-axis indicates dose (MET·min/week) and the *y*-axis indicates the model-predicted effect size (Hedges’ *g*). Solid black lines represent the estimated non-linear dose–response functions and dashed black lines represent the 95% credible intervals; the horizontal grey dashed line denotes the null effect (*g* = 0). Red vertical dashed lines and red points indicate modality-specific predictions at key dose nodes (annotated with the corresponding dose values). The shaded background gradient is provided to facilitate visual interpretation across dose ranges.

Figure 7 presents dose–response curves by exercise modality. Aerobic exercise, resistance training, and water-based exercise exhibited an inverted U-shaped relationship, reaching peak predicted effects at 554, 666, and 1,616 METs-min/week, respectively (aerobic: *g* = 0.64, 95% CrI [0.52, 0.76]; posterior SD = 0.06; resistance: *g* = 1.29, [1.21, 1.37]; posterior SD = 0.04; water-based: *g* = 1.73, [1.56, 1.90]; posterior SD = 0.09). For CE and BT, predicted effects increased with dose across the data-supported range; therefore, these results are best interpreted as a stable positive association rather than overinterpreting the exact peak location or marginal gains at the high-dose end. In contrast, the predicted effect for HIIT was small in magnitude, and its 95% CrI included zero across most of the dose range, indicating substantial uncertainty and insufficient evidence for a stable, dose-specific improvement.

### Evidence-informed dose guidance at WHO-recommended activity levels

**Figure 8:**
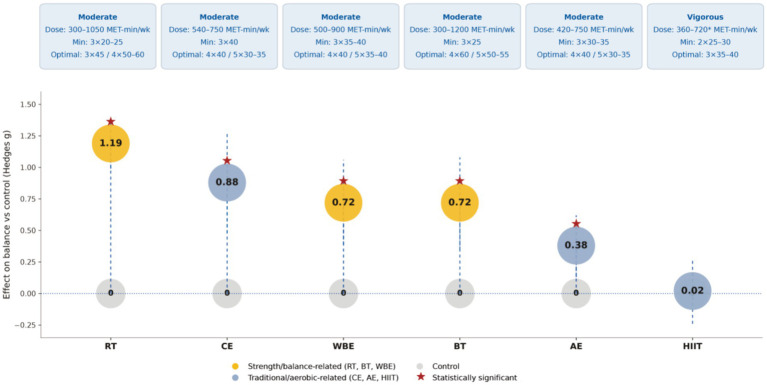
Overall effects of different exercise modalities on balance and illustrative recommended doses. The *x*-axis shows intervention type (RT, CE, WBE, BT, AE, and HIIT), and the *y*-axis shows the effect size relative to the control group (Hedges’ *g*). Colored circles indicate the pooled effect estimates for each modality (*g* values shown inside the circles), and dashed vertical lines represent uncertainty intervals; the horizontal dashed line denotes the null effect (*g* = 0). The boxes above summarize illustrative recommended exercise doses for each modality, including intensity level, MET·min/week range, and minimum/optimal combinations of frequency and session duration. Colors distinguish strength/balance-related training (RT, BT, WBE) from traditional/aerobic-related training (CE, AE, HIIT). Red stars indicate statistical significance (*p* < 0.05).

Model-predicted effects for each intervention at prespecified WHO-recommended dose nodes (600, 1,200, and 1800 MET-min/week) are reported in Supplementary File 6, together with posterior SDs and 95% credible intervals (CrIs). Posterior rankograms for dose-specific nodes (intervention × dose) are also provided to support interpretation of the dose–response relationship and model selection. Figure 8 synthesizes these estimates and presents evidence-informed considerations for exercise dosing in practice.

### Small-study effects and publication bias

**Figure 9:**
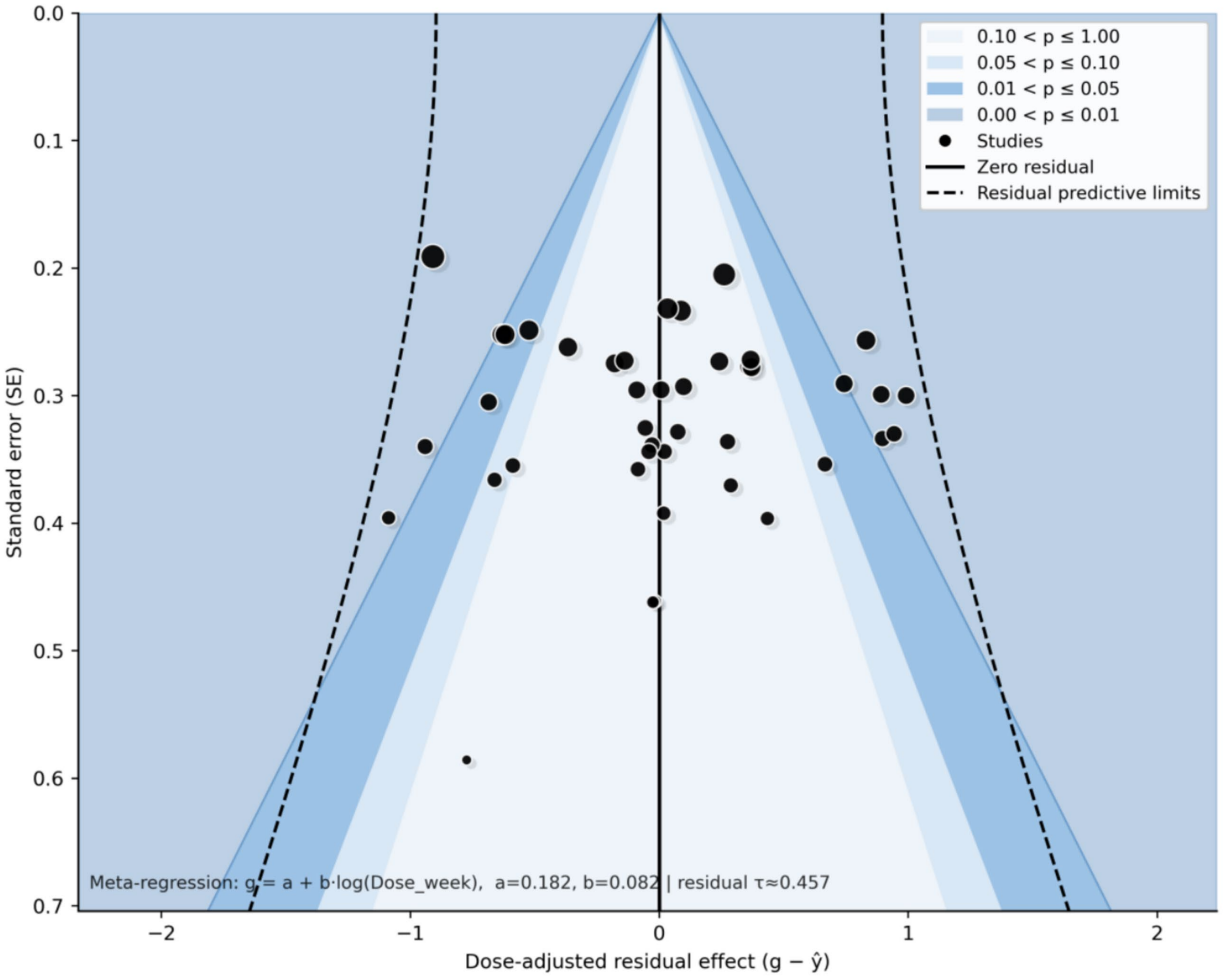
Dose-adjusted contour-enhanced funnel plot. The *x*-axis shows the dose-adjusted residual effect (*g* − ŷ) and the *y*-axis shows the standard error (SE). Each black point represents an individual study; the solid vertical line denotes the zero-residual line, and dashed lines indicate the residual predictive limits. Shaded regions correspond to different levels of statistical significance (*p*-value contours). The residuals appear approximately symmetrically distributed around the zero-residual line, providing no clear visual indication of small-study effects or publication bias; however, visual assessment alone cannot exclude residual bias.

Small-study effects were visually assessed using a dose-adjusted contour-enhanced funnel plot of meta-regression residuals (*x*-axis: g − ŷ; *y*-axis: standard error). The points were broadly distributed around the zero-residual line, concentrated mainly at SE ≈ 0.20–0.40, and largely within the residual predictive limits, with no obvious one-sided gap or marked asymmetry (Figure 9). Overall, visual inspection did not indicate a clear signal of small-study effects, suggesting that the main findings are unlikely to be driven by small-study effects.

## Discussion

This study integrates Bayesian random-effects network meta-analysis with model-based network meta-analysis (MBNMA) to synthesize, on a unified dose scale (MET-min/week), both comparative effects across exercise modalities and modality-specific dose–response relationships for balance recovery after stroke. Balance impairment is a prevalent and clinically meaningful consequence of stroke, closely linked to falls, independent ambulation, and activities of daily living (31). The Berg Balance Scale (BBS) was selected as the primary outcome because it captures global static and dynamic postural control across functional tasks and is sufficiently reliable and responsive to support cross-trial comparison (32). By harmonizing dose and jointly modeling modality and dose, the present synthesis aims to support a prescription-oriented interpretation of the literature—what appears to work, at approximately what weekly dose range, and under what evidential constraints.

At the modality level, resistance training (RT) demonstrated the largest pooled improvement and a high probability of ranking among the most effective approaches, whereas high-intensity interval training (HIIT) produced smaller and imprecise pooled estimates (33). This pattern is compatible with core contributors to post-stroke balance limitation, including reduced lower-limb strength and power, delayed corrective responses, and impaired coordination across the trunk–pelvis–lower-limb chain (34, 35). RT may plausibly address these constraints by improving load acceptance and force-generation capacity at key joints (notably knee and ankle), thereby enlarging the feasible control space for center-of-mass regulation during functional tasks sampled by the BBS (36, 37). In contrast, HIIT effectiveness for balance may depend strongly on whether the intended intensity is achieved and sustained, which is influenced by prescription structure, monitoring, and individual tolerance (38, 39). Given substantial post-stroke heterogeneity in fatigue sensitivity and comorbidity burden, variability in achieved intensity and dose fidelity may plausibly contribute to the uncertainty observed in pooled HIIT effects (40, 41). Accordingly, interpretation should be evidence-graded: current certainty does not support routine first-line recommendation of HIIT for balance improvement, while any protocol-specific benefit—if suggested in individual trials—should be described with explicit boundary conditions (patient selection, monitoring strategy, and progression criteria) to reconcile protocol-level effects with the overall network estimate (42).

Across modalities, the overall dose–response relationship was non-linear, with predicted benefit increasing up to approximately 1,200 MET-min/week and then showing attenuation/decline, with a minimum effective dose around 270 MET-min/week (23). Clinically, these findings support two pragmatic implications for stroke rehabilitation. First, a relatively low minimum effective dose is consistent with initiating training at attainable weekly volumes in deconditioned patients and progressing as tolerated, rather than assuming that only high weekly volumes are clinically meaningful (10, 24). Second, the non-monotonic pattern argues against a “more is better” heuristic and instead supports dose progression constrained by movement quality, fatigue response, and safety as weekly volume increases (9, 13). Mechanistic explanations for diminishing returns at higher doses should be considered hypothesis-generating. Post-stroke metabolic inefficiency and early fatigue may degrade movement quality and increase compensatory strategies; if task execution quality declines, additional volume could contribute less to the sensorimotor relearning processes relevant to balance (43, 44). In practice, this interpretation is more compatible with a progressive prescription conditioned on fatigue and task quality (e.g., recovery scheduling and stopping rules) than with volume escalation alone (38). Collectively, the evidence supports a pragmatic “start low and progress by quality” approach in stroke rehabilitation: initiate at feasible weekly doses, monitor fatigue and task execution quality, and individualize progression based on recovery capacity and safety. WHO reference points (600/1200/1800 MET-min/week) can facilitate communication and standardization on a unified dose scale, but they were developed for general adult populations and are used here only as anchors; they should not be interpreted as stroke-specific neurorehabilitation thresholds or absolute clinical cut-offs (45).

Dose–response shapes varied by modality. Aerobic training, RT, and aquatic training demonstrated inverted U-shaped patterns, whereas traditional Chinese exercise (CE) and balance training (BT) remained positive within the evidence-supported dose range (16, 46). This heterogeneity suggests that balance gains may depend more on whether the training stimulus targets dominant impairments than on maximizing total dose (47, 48). Aerobic training may support endurance and trunk involvement relevant to postural control, but higher doses may introduce fatigue-related decrements that offset gains in balance task performance (49, 50). For RT, insufficient recovery under higher loads may similarly compromise the refined control required for balance-related tasks (40, 41). Aquatic training may facilitate practice by reducing mechanical loading while providing multidirectional sensory inputs, yet tolerability and access may constrain high-dose implementation (51, 52). In contrast, CE and BT emphasize task-specific weight shifting, trunk control, and coordination challenges that may sustain benefit across studied doses (17, 53). Nevertheless, evidence becomes sparse at higher doses and uncertainty widens; therefore, inferences should be restricted to dose ranges represented in the available trials and should not be extrapolated beyond the evidence base (23, 24). For HIIT, when credible intervals across most dose ranges include no effect, current evidence does not support a stable dose-specific benefit; this pattern is more consistent with protocol heterogeneity and limitations in achieving effective intensity under safety constraints than with a definitive absence of effect (42).

Several considerations temper interpretation. First, the network is star-shaped and anchored to a common comparator, limiting formal inconsistency assessment and increasing model dependence of rankings; therefore, rankings should be interpreted as descriptive summaries and weighed against effect sizes, uncertainty, and certainty of evidence. Second, although we standardized dose to MET-min/week to support cross-trial synthesis, dose in rehabilitation trials may still reflect heterogeneous combinations of frequency, duration, and intensity; clinical translation should prioritize feasibility and quality of execution rather than treating any single weekly dose value as prescriptive. Third, risk of bias and small samples remain important constraints in exercise trials; our RoB-based sensitivity analysis excluding overall high-risk trials supports the robustness of key directional conclusions, but uncertainty increases where evidence becomes sparse, particularly at higher doses. Finally, the evidence base is dominated by chronic-phase stroke and short-to-moderate intervention durations, which limits generalizability to acute stroke or severely impaired populations; future trials should prospectively test dose progression strategies in these groups with explicit reporting of achieved intensity, adherence, adverse events, and movement quality.

### Practical applications

These results support a prescription-oriented approach to exercise after stroke: selecting modalities based on comparative network estimates while guiding starting dose and progression using dose–response relationships on a unified MET-min/week scale (10). The estimated minimum effective dose (~270 MET-min/week) can inform low-dose initiation, particularly for patients with limited baseline capacity, with progression constrained by fatigue response, safety monitoring, adherence, and movement quality (9). WHO benchmarks may facilitate standardized communication on a common dose metric; however, they were developed for general adult populations and are used here only as communication anchors rather than stroke-specific rehabilitation thresholds. Practical recommendations should remain within dose ranges covered by the available evidence, especially given widening uncertainty at higher doses. Model-predicted effects and uncertainty at selected reference doses are provided in the Supplementary materials, and Figure 7 summarizes dose anchors to support individualized adjustment and follow-up monitoring.

### Limitations

Several limitations merit consideration. First, dose estimation in MET-min/week depends on reporting quality of intensity, frequency, and duration; incomplete intensity reporting and heterogeneous conversion assumptions may introduce dose misclassification. Second, discretization of continuous dose was used to support split-NMA connectivity and visualization, which may introduce approximation error; we therefore report discretization robustness checks in the Supplementary materials. Third, evidence coverage is uneven across modalities and limited at higher doses, leading to wider credible intervals and reduced precision in identifying the location of any peak and the magnitude of attenuation/decline; high-dose inferences should therefore be confined to evidence-supported ranges. Fourth, although focusing on the BBS improves cross-trial comparability, generalization to other balance domains (e.g., dynamic stability metrics, real-world falls, and gait-related endpoints) should be cautious. Finally, small-study effects were assessed visually using dose-adjusted residual funnel plots; the absence of marked asymmetry does not fully exclude residual bias or selective reporting.

## Conclusion

By combining Bayesian network meta-analysis with MBNMA on a unified dose scale, this study compared exercise modalities for improving balance after stroke and characterized dose–response relationships within the available evidence base. Low-to-moderate doses were associated with credible improvements, whereas the overall dose–response pattern was non-linear and high-dose inferences were more uncertain due to limited evidence coverage. At the modality level, resistance training showed the largest pooled benefit versus control; however, rankings should be interpreted as descriptive and model-dependent given the star-shaped network structure and predominantly low-to-moderate certainty evidence. Modality-specific heterogeneity indicates that exercise prescription should jointly consider modality selection and dose progression guided by tolerance and movement quality rather than pursuing higher volume alone. These conclusions are based largely on small trials with short intervention durations and predominantly chronic-phase stroke populations; generalization to acute stroke or severely impaired populations should be made cautiously and requires dedicated trials.

## Data Availability

The datasets presented in this study can be found in online repositories. The names of the repository/repositories and accession number(s) can be found in the article/Supplementary material.

## References

[1] Feigin VL Brainin M Norrving B Martins SO Pandian J Lindsay P . World stroke organization: global stroke fact sheet 2025. Int J Stroke. (2025) 20:132–44. doi: 10.1177/17474930241308142, 39635884 PMC11786524

[2] Tyson SF Hanley M Chillala J Selley A Tallis RC. Balance disability after stroke. Phys Ther. (2006) 86:30–8. doi: 10.1093/ptj/86.1.30, 16386060

[3] Weerdesteyn V de Niet M van Duijnhoven HJR Geurts ACH. Falls in individuals with stroke. J Rehabil Res Dev. (2008) 45:1195–213. doi: 10.1682/JRRD.2007.09.0145, 19235120

[4] Goh HT Nadarajah M Hamzah B Varadan P Tan MP. Falls and fear of falling after stroke: a case-control study. PM R. (2016) 8:1173–80. doi: 10.1016/j.pmrj.2016.05.012, 27268565

[5] Schmid AA Arnold SE Jones VA Ritter MJ Sapp SA Van Puymbroeck M. Fear of falling in people with chronic stroke. Am J Occup Ther. (2015) 69:6903350020p1–5. doi: 10.5014/ajot.2015.016253, 25871606 PMC4453042

[6] Schinkel-Ivy A Inness EL Mansfield A. Relationships between fear of falling, balance confidence, and control of balance, gait, and reactive stepping in individuals with sub-acute stroke. Gait Posture. (2016) 43:154–9. doi: 10.1016/j.gaitpost.2015.09.015, 26482234 PMC5045898

[7] Caspersen CJ Powell KE Christenson GM. Physical activity, exercise, and physical fitness: definitions and distinctions for health-related research. Public Health Rep. (1985) 100:126–31. 3920711 PMC1424733

[8] World Health Organization. Physical Activity (Fact Sheet). Geneva, Switzerland: World Health Organization. (2024).

[9] Winstein CJ Stein J Arena R Bates B Cherney LR Cramer SC . Guidelines for adult stroke rehabilitation and recovery. Stroke. (2016) 47:e98–e169. doi: 10.1161/STR.0000000000000098, 27145936

[10] Billinger SA Arena R Bernhardt J Eng JJ Franklin BA Johnson CM . Physical activity and exercise recommendations for stroke survivors: a statement for healthcare professionals from the American Heart Association/American Stroke Association. Stroke. (2014) 45:2532–53. doi: 10.1161/STR.0000000000000022, 24846875

[11] Gordon NF Gulanick M Costa F Fletcher G Franklin BA Roth EJ . Physical activity and exercise recommendations for stroke survivors: an American Heart Association scientific statement. Circulation. (2004) 109:2031–41. doi: 10.1161/01.CIR.0000126280.65777.A4 15117863

[12] World Health Organization. WHO Guidelines on Physical Activity and Sedentary Behaviour. Geneva: WHO (2020). 33369898

[13] Bull FC Al-Ansari SS Biddle S Borodulin K Buman MP Cardon G . World Health Organization 2020 guidelines on physical activity and sedentary behaviour. Br J Sports Med. (2020) 54:1451–62. doi: 10.1136/bjsports-2020-102955, 33239350 PMC7719906

[14] Winstein C Kim B Kim S Martinez C Schweighofer N. Dosage Matters. Stroke. (2019) 50:1831–7. doi: 10.1161/STROKEAHA.118.023603, 31164067 PMC12718071

[15] Arienti C Lazzarini SG Pollock A Negrini S. Rehabilitation interventions to improve balance following stroke: an overview of systematic reviews. PLoS One. (2019) 14:e0219781. doi: 10.1371/journal.pone.0219781, 31323068 PMC6641159

[16] van Duijnhoven HJR Heeren A Peters MAM Veerbeek JM Kwakkel G Geurts ACH . Effects of exercise therapy on balance capacity in chronic stroke: systematic review and meta-analysis. Stroke. (2016) 47:2603–10. doi: 10.1161/STROKEAHA.116.013839, 27633021

[17] Lubetzky-Vilnai A Kartin D. The effect of balance training on balance performance in individuals poststroke: a systematic review. J Neurol Phys Ther. (2010) 34:127–37. doi: 10.1097/NPT.0b013e3181ef764d, 20716987

[18] Denissen S Staring W Kunkel D Pickering RM Lennon S Geurts ACH . Interventions for preventing falls in people after stroke. Cochrane Database Syst Rev. (2019) 2019:CD008728. doi: 10.1002/14651858.CD008728.pub3, 31573069 PMC6770464

[19] Denissen S Staring W Kunkel D Pickering RM Lennon S Geurts ACH . Interventions for preventing falls in people after stroke. Stroke. (2020) 51:e47–8. doi: 10.1161/STROKEAHA.119.028157, 32008458

[20] Jørgensen L Engstad T Jacobsen BK. Higher incidence of falls in long-term stroke survivors than in population controls: depressive symptoms predict falls after stroke. Stroke. (2002) 33:542–7. doi: 10.1161/hs0202.102375, 11823667

[21] Salanti G. Indirect and mixed-treatment comparison, network, or multiple-treatments meta-analysis: many names, many benefits, many concerns for the next generation evidence synthesis tool. Res Synth Methods. (2012) 3:80–97. doi: 10.1002/jrsm.1037, 26062083

[22] Lu G Ades AE. Mixed treatment comparisons: combination of direct and indirect evidence. Stat Med. (2004) 23:3105–24. doi: 10.1002/sim.1875 15449338

[23] Mawdsley D Bennetts M Dias S Boucher M Welton NJ. Model-based network Meta-analysis: a framework for evidence synthesis of clinical trial data. CPT Pharmacometrics Syst Pharmacol. (2016) 5:393–401. doi: 10.1002/psp4.12091, 27479782 PMC4999602

[24] Pedder H Dias S Boucher M Bennetts M Mawdsley D Welton NJ. Methods to assess evidence consistency in dose-response model based network Meta-analysis. Stat Med. (2022) 41:625–44. doi: 10.1002/sim.9270, 34866221

[25] Dias S Welton NJ Sutton AJ Ades AE. NICE DSU Technical Support Document 2: A Generalised linear Modelling Framework for Pairwise and Network meta-Analysis of Randomised Controlled Trials.National Institute for Health and Clinical Excellence (2011). 27466657

[26] Page MJ McKenzie JE Bossuyt PM Boutron I Hoffmann TC Mulrow CD . The PRISMA 2020 statement: an updated guideline for reporting systematic reviews. BMJ. (2021) 372:n71. doi: 10.1136/bmj.n71 33782057 PMC8005924

[27] R Core Team. R: A Language and Environment for Statistical Computing. Vienna, Austria: R Foundation for Statistical Computing (2020).

[28] Viechtbauer W. Conducting meta-analyses in R with the metafor package. J Stat Softw. (2010) 36:1–48. doi: 10.18637/jss.v036.i03

[29] Salanti G Ades AE Ioannidis JPA. Graphical methods and numerical summaries for presenting results from multiple-treatment meta-analysis: an overview and tutorial. J Clin Epidemiol. (2011) 64:163–71. doi: 10.1016/j.jclinepi.2010.03.016, 20688472

[30] Wickham H. ggplot2: Elegant Graphics for Data Analysis. New York: Springer (2016).

[31] Hugues A Di Marco J Janiaud P Xue Y Pires J Khademi H . Efficiency of physical therapy on postural imbalance after stroke: study protocol for a systematic review and meta-analysis. BMJ Open. (2017) 7:e013348. doi: 10.1136/bmjopen-2016-013348 PMC5293873 28137928

[32] Tamura S Miyata K Kobayashi S Takeda R Iwamoto H. The minimal clinically important difference in Berg balance scale scores among patients with early subacute stroke: a multicenter, retrospective, observational study. Top Stroke Rehabil. (2022) 29:423–9. doi: 10.1080/10749357.2021.1943800 34169808

[33] Berg KO Wood-Dauphinee SL Williams JI Maki B. Measuring balance in the elderly: validation of an instrument. Can J Public Health. (1992) 83:S7–S11. 1468055

[34] Blum L Korner-Bitensky N. Usefulness of the Berg balance scale in stroke rehabilitation: a systematic review. Phys Ther. (2008) 88:559–66. doi: 10.2522/ptj.20070205, 18292215

[35] Stevenson TJ. Detecting change in patients with stroke using the Berg balance scale. Aust J Physiother. (2001) 47:29–38. doi: 10.1016/S0004-9514(14)60296-8 11552860

[36] Hiengkaew V Jitaree K Chaiyawat P. Minimal detectable changes of the Berg balance scale and other measures in chronic stroke. Arch Phys Med Rehabil. (2012) 93:1206–10. doi: 10.1016/j.apmr.2012.01.014 22502805

[37] Hunnicutt JL Gregory CM. Skeletal muscle changes following stroke: a systematic review and comparison to healthy individuals. Top Stroke Rehabil. (2017) 24:463–71. doi: 10.1080/10749357.2017.1292720, 28251861 PMC5801663

[38] Azzollini V Dalise S Chisari C. How does stroke affect skeletal muscle? State of the art and rehabilitation perspective. Front Neurol. (2021) 12:797559. doi: 10.3389/fneur.2021.797559, 35002937 PMC8733480

[39] Brough LG Kautz SA Neptune RR. Muscle contributions to pre-swing biomechanical tasks influence swing leg mechanics in individuals post-stroke during walking. J Neuroeng Rehabil. (2022) 19. Published 2022 Jun 3:55. doi: 10.1186/s12984-022-01029-z, 35659252 PMC9166530

[40] Flansbjer U-B Downham D Lexell J. Progressive resistance training after stroke: effects on muscle strength, muscle tone, gait performance and perceived participation. J Rehabil Med. (2008) 40:42–8. doi: 10.2340/16501977-0129 18176736

[41] Dorsch S Ada L Alloggia D. Progressive resistance training increases strength after stroke but this may not carry over to activity: a systematic review. J Physiother. (2018) 64:84–90. doi: 10.1016/j.jphys.2018.02.012 29602748

[42] Gjellesvik TI Becker F Tjønna AE Indredavik B Nilsen H Brurok B . Effects of high-intensity interval training after stroke (HIIT-stroke study): a multicenter randomized controlled trial. Arch Phys Med Rehabil. (2020) 101:939–47. doi: 10.1016/j.apmr.2020.02.006 32145280

[43] Alghamdi I Ariti C Williams A Wood E Hewitt J. Prevalence of fatigue after stroke: a systematic review and meta-analysis. Eur Stroke J. (2021) 6:319–32. doi: 10.1177/23969873211047681, 35342803 PMC8948505

[44] Larsson P Bidonde J Olsen U Gay CL Lerdal A Ursin M . Association of post-stroke fatigue with physical activity and physical fitness: a systematic review and meta-analysis. Int J Stroke. (2023) 18:1063–70. doi: 10.1177/17474930231152132, 36622013 PMC11044520

[45] Ainsworth BE Haskell WL Herrmann SD Meckes N Bassett DR Tudor-Locke C . 2011 compendium of physical activities: a second update of codes and MET values. Med Sci Sports Exerc. (2011) 43:1575–81. doi: 10.1249/MSS.0b013e31821ece12, 21681120

[46] Saunders DH Sanderson M Hayes S Johnson L Kramer S Carter DD . Physical fitness training for stroke patients. Cochrane Database Syst Rev. (2020). doi: 10.1002/14651858.CD003316.pub7 PMC7083515 32196635

[47] Kleim JA Jones TA. Principles of experience-dependent neural plasticity: implications for rehabilitation after brain damage. J Speech Lang Hear Res. (2008) 51:S225–39. doi: 10.1044/1092-4388(2008/018), 18230848

[48] Hubbard IJ Parsons MW Neilson C Carey LM. Task-specific training: evidence for and translation to clinical practice. Occup Ther Int. (2009) 16:175–89. doi: 10.1002/oti.275, 19504501

[49] Harmon EY Melewski M Provost D Sonagere MB. Safety and benefits of moderate to high intensity aerobic exercise during the subacute phase of stroke: a systematic review and meta-analysis. Arch Rehabil Res Clin Transl. (2025):100551. doi: 10.1016/j.arrct.2025.100551 41834808 PMC12988557

[50] Wu S Chen J Wang S Jiang M Wang X Wen Y. Effect of tai chi exercise on balance function of stroke patients: a Meta-analysis. Med Sci Monit Basic Res. (2018) 24:210–5. Published 2018 Dec 3. doi: 10.12659/MSMBR.911951, 30504762 PMC6289026

[51] Iliescu AM McIntyre A Wiener J Iruthayarajah J Lee A Caughlin S . Aquatic therapy for mobility, balance and functional independence after stroke: systematic review and meta-analysis. Clin Rehabil. (2020) 34:56–68. doi: 10.1177/0269215519880955 31625407

[52] Nascimento LR Flores LC de Menezes KKP Teixeira-Salmela LF. Water-based exercises for improving walking speed, balance, and strength after stroke: a systematic review with meta-analyses of randomized trials. Physiotherapy. (2020) 107:100–10. doi: 10.1016/j.physio.2019.10.002, 32026809

[53] Lyu D Lyu X Zhang Y Ren Y Yang F Zhou L . Tai chi for stroke rehabilitation: a systematic review and Meta-analysis of randomized controlled trials. Front Physiol. (2018) 9:983. doi: 10.3389/fphys.2018.00983, 30090071 PMC6068268

