# Supplementary material for: Optimal exercise prescription for balance after stroke: a Bayesian dose–response network meta-analysis
Source: Front Neurol. 2026 Apr 13;17:1804224. doi: 10.3389/fneur.2026.1804224 (PMC13110969; doi:10.3389/fneur.2026.1804224)
Supplement: Supplementary file 1 [file Supplementary_file_1.DOCX]

**Supplementary file 1: Search strategy**

# MEDLINE (PubMed)

***Last searched: 2025-12-31***

1. Stroke[Mesh]

2. Brain Ischemia[Mesh] OR Cerebral Infarction[Mesh]

3. Intracranial Hemorrhages[Mesh] OR Cerebral Hemorrhage[Mesh] OR Intracerebral Hemorrhage[tiab]

4. stroke*[tiab] OR poststroke[tiab] OR "post-stroke"[tiab] OR "cerebrovascular accident*"[tiab] OR CVA[tiab] OR "brain infarct*"[tiab] OR "cerebral infarct*"[tiab] OR "ischemic stroke"[tiab] OR "ischaemic stroke"[tiab] OR "hemorrhagic stroke"[tiab] OR "haemorrhagic stroke"[tiab]

5. #1 OR #2 OR #3 OR #4

6. Postural Balance[Mesh]

7. balance[tiab] OR "balance function"[tiab] OR "balance ability"[tiab] OR "postural control"[tiab] OR "postural stabil*"[tiab] OR "postural sway"[tiab] OR "dynamic balance"[tiab] OR "static balance"[tiab]

8. "Berg Balance Scale"[tiab] OR BBS[tiab] OR "Timed Up and Go"[tiab] OR TUG[tiab] OR "functional reach"[tiab] OR "limit* of stability"[tiab]

9. #6 OR #7 OR #8

10. Exercise Therapy[Mesh] OR Exercise[Mesh] OR Rehabilitation[Mesh]

11. Resistance Training[Mesh] OR "resistance training"[tiab] OR "strength training"[tiab] OR "resistance exercise"[tiab] OR "progressive resistance"[tiab] OR "weight training"[tiab]

12. "aerobic training"[tiab] OR "aerobic exercise"[tiab] OR "endurance training"[tiab]

13. "balance training"[tiab] OR "postural training"[tiab] OR "task-oriented training"[tiab] OR "task oriented training"[tiab]

14. "high-intensity interval training"[tiab] OR "high intensity interval training"[tiab] OR HIIT[tiab] OR "interval training"[tiab]

15. Aquatic Therapy[Mesh] OR "aquatic exercise"[tiab] OR "water-based exercise"[tiab] OR "water based exercise"[tiab] OR hydrotherapy[tiab]

16. Tai Ji[Mesh] OR Qigong[Mesh] OR "tai chi"[tiab] OR taiji[tiab] OR qigong[tiab] OR "traditional Chinese exercise"[tiab]

17. #10 OR #11 OR #12 OR #13 OR #14 OR #15 OR #16

18. randomized controlled trial[pt] OR controlled clinical trial[pt] OR randomi?ed[tiab] OR randomly[tiab] OR trial[tiab] OR placebo[tiab] OR RCT[tiab]

19. animals[mh] NOT humans[mh]

20. #18 NOT #19

21. #5 AND #9 AND #17 AND #20

# Embase (Embase.com)

**Last searched:** 2025-12-31

1. 'stroke'/exp OR 'cerebrovascular accident'/exp OR 'brain ischemia'/exp OR 'cerebral infarction'/exp OR 'intracerebral hemorrhage'/exp

2. stroke*:ti,ab OR poststroke:ti,ab OR "post-stroke":ti,ab OR "cerebrovascular accident*":ti,ab OR CVA:ti,ab OR "ischemic stroke":ti,ab OR "haemorrhagic stroke":ti,ab OR "hemorrhagic stroke":ti,ab

3. #1 OR #2

4. 'postural balance'/exp OR 'posture control'/exp

5. balance:ti,ab OR "postural control":ti,ab OR "postural stabil*":ti,ab OR "dynamic balance":ti,ab OR "static balance":ti,ab OR "postural sway":ti,ab

6. "Berg Balance Scale":ti,ab OR BBS:ti,ab OR "Timed Up and Go":ti,ab OR TUG:ti,ab OR "functional reach":ti,ab OR "limit* of stability":ti,ab

7. #4 OR #5 OR #6

8. 'exercise therapy'/exp OR 'exercise'/exp OR 'rehabilitation'/exp

9. 'resistance training'/exp OR 'strength training'/exp OR "resistance training":ti,ab OR "strength training":ti,ab OR "weight training":ti,ab

10. 'aerobic exercise'/exp OR "aerobic training":ti,ab OR "endurance training":ti,ab

11. "balance training":ti,ab OR "postural training":ti,ab OR "task oriented training":ti,ab OR "task-oriented training":ti,ab

12. 'high intensity interval training'/exp OR "high intensity interval training":ti,ab OR HIIT:ti,ab OR "interval training":ti,ab

13. 'aquatic therapy'/exp OR "aquatic exercise":ti,ab OR "water based":ti,ab OR "water-based":ti,ab OR hydrotherapy:ti,ab

14. 'tai chi'/exp OR 'qigong'/exp OR "tai chi":ti,ab OR taiji:ti,ab OR qigong:ti,ab OR "traditional Chinese exercise":ti,ab

15. #8 OR #9 OR #10 OR #11 OR #12 OR #13 OR #14

16. 'randomized controlled trial'/de OR 'randomization'/de OR 'single blind procedure'/de OR 'double blind procedure'/de

17. random*:ti,ab OR trial:ti,ab OR placebo:ti,ab OR RCT:ti,ab

18. #16 OR #17

19. #18 AND [humans]/lim

20. #3 AND #7 AND #15 AND #19

# Web of Science Core Collection

**Last searched:** 2025-12-31

1. TS=(stroke OR stroke* OR poststroke OR "post-stroke" OR "cerebrovascular accident*" OR CVA OR "brain infarct*" OR "cerebral infarct*" OR "brain ischem*" OR "ischemic stroke" OR "ischaemic stroke" OR "intracerebral hemorrhag*" OR "cerebral hemorrhag*" OR "hemorrhagic stroke" OR "haemorrhagic stroke")

2. TS=(balance OR "postural control" OR "postural stabil*" OR "postural sway" OR "dynamic balance" OR "static balance" OR "Berg Balance Scale" OR BBS OR "Timed Up and Go" OR TUG OR "functional reach" OR "limit* of stability")

3. TS=(exercise OR "exercise therapy" OR rehabilitation OR "aerobic training" OR "aerobic exercise" OR "endurance training" OR "resistance training" OR "strength training" OR "weight training" OR "balance training" OR "postural training" OR "high intensity interval training" OR "high-intensity interval training" OR HIIT OR "interval training" OR "aquatic exercise" OR "water-based exercise" OR hydrotherapy OR "aquatic therapy" OR "tai chi" OR taiji OR qigong OR "traditional Chinese exercise")

4. TS=(random* OR trial OR trials OR "controlled trial" OR RCT OR placebo)

5. #1 AND #2 AND #3 AND #4

# Scopus

**Last searched:** 2025-12-31

1. TITLE-ABS-KEY(stroke OR stroke* OR poststroke OR "post-stroke" OR "cerebrovascular accident*" OR CVA OR "brain infarct*" OR "cerebral infarct*" OR "brain ischem*" OR "ischemic stroke" OR "ischaemic stroke" OR "intracerebral hemorrhag*" OR "cerebral hemorrhag*" OR "hemorrhagic stroke" OR "haemorrhagic stroke")

2. TITLE-ABS-KEY(balance OR "postural control" OR "postural stabil*" OR "postural sway" OR "dynamic balance" OR "static balance" OR "Berg Balance Scale" OR BBS OR "Timed Up and Go" OR TUG OR "functional reach" OR "limit* of stability")

3. TITLE-ABS-KEY(exercise OR "exercise therapy" OR rehabilitation OR "aerobic training" OR "aerobic exercise" OR "endurance training" OR "resistance training" OR "strength training" OR "weight training" OR "balance training" OR "postural training" OR "high intensity interval training" OR "high-intensity interval training" OR HIIT OR "interval training" OR "aquatic exercise" OR "water-based exercise" OR hydrotherapy OR "aquatic therapy" OR "tai chi" OR taiji OR qigong OR "traditional Chinese exercise")

4. TITLE-ABS-KEY(random* OR trial OR trials OR "controlled trial" OR RCT OR placebo)

5. #1 AND #2 AND #3 AND #4

# Cochrane Central Register of Controlled Trials (CENTRAL)

**Last searched:** 2025-12-31

1. (stroke OR "cerebrovascular accident*" OR poststroke OR "post-stroke" OR "brain infarct*" OR "cerebral infarct*" OR "ischemic stroke" OR "intracerebral hemorrhag*"):ti,ab,kw

2. (balance OR "postural control" OR "postural stabil*" OR "Berg Balance Scale" OR BBS OR "Timed Up and Go" OR TUG):ti,ab,kw

3. (exercise OR "exercise therapy" OR rehabilitation OR "aerobic training" OR "resistance training" OR "strength training" OR "balance training" OR HIIT OR "high intensity interval training" OR "aquatic exercise" OR "water-based exercise" OR hydrotherapy OR "tai chi" OR taiji OR qigong):ti,ab,kw

4. #1 AND #2 AND #3

# Chinese databases

Databases: CNKI, Wanfang, and VIP.

## CNKI (China National Knowledge Infrastructure)

**Last searched:** 2025-12-31

1. Topic= (Stroke OR Cerebral Apoplexy OR Cerebrovascular Accident OR Ischemic Stroke OR Cerebral Infarction OR Cerebral Infarction OR Cerebral Hemorrhage)
2. Topic= (Balance OR Balance Function OR Postural Control OR Postural Stability OR "Berg Balance Scale" OR BBS OR Dynamic Balance OR Static Balance)
3. Topic= (Exercise OR Exercise OR Training OR Rehabilitation Training OR Aerobic OR Aerobic Exercise OR Resistance OR Resistance Training OR Strength Training OR Balance Training OR High-Intensity Interval Training OR HIIT OR Water Training OR Hydrotherapy OR Tai Chi OR Qigong OR Traditional Gongfa)
4. Topic= (Randomized Controlled Trial OR Random OR Control OR RCT OR Clinical Trial) 5. #1 AND #2 AND #3 AND #4

## Wanfang Data

**Last searched:** 2025-12-31

## 1.Topic= (Stroke OR Cerebral Apoplexy OR Cerebrovascular Accident OR Ischemic Stroke OR Cerebral Infarction OR Cerebral Infarction OR Cerebral Hemorrhage)

## 2. Topic= (Balance OR Balance Function OR Postural Control OR Postural Stability OR "Berg Balance Scale" OR BBS OR Dynamic Balance OR Static Balance)

## 3. Topic= (Exercise OR Exercise OR Training OR Rehabilitation Training OR Aerobic OR Aerobic Exercise OR Resistance OR Resistance Training OR Strength Training OR Balance Training OR High-Intensity Interval Training OR HIIT OR Water Training OR Hydrotherapy OR Tai Chi OR Qigong OR Traditional Gongfa)

## 4. Topic= (Randomized Controlled Trial OR Random OR Control OR RCT OR Clinical Trial) 5. #1 AND #2 AND #3 AND #4VIP (Chinese Scientific Journals Database)

**Last searched:** 2025-12-31

# 1.Topic= (Stroke OR Cerebral Apoplexy OR Cerebrovascular Accident OR Ischemic Stroke OR Cerebral Infarction OR Cerebral Infarction OR Cerebral Hemorrhage)

# 2. Topic= (Balance OR Balance Function OR Postural Control OR Postural Stability OR "Berg Balance Scale" OR BBS OR Dynamic Balance OR Static Balance)

# 3. Topic= (Exercise OR Exercise OR Training OR Rehabilitation Training OR Aerobic OR Aerobic Exercise OR Resistance OR Resistance Training OR Strength Training OR Balance Training OR High-Intensity Interval Training OR HIIT OR Water Training OR Hydrotherapy OR Tai Chi OR Qigong OR Traditional Gongfa)

# 4. Topic= (Randomized Controlled Trial OR Random OR Control OR RCT OR Clinical Trial) 5. #1 AND #2 AND #3 AND #4Additional searching

Reference lists of included studies and relevant systematic reviews were screened (backward citation tracking) to identify additional eligible trials.

**Supplementary file 2: Key assumptions of Network Meta-Analysis**

There are three key assumptions to conduct a Network Meta-Analysis (NMA): (1) network connectivity, (2) consistency in the data, and (3) transitivity

***Connectivity***

Connectivity is a key assumption in NMA which if deemed insufficient (i.e., due to lack of direct comparators) can lead to low statistical power and misleading results. In our study, we assessed connectivity of the network at both treatment and agent levels visually and found no evidence of unconnectedness on either network (Supplementary Figure 1 and Supplementary Figure 2).


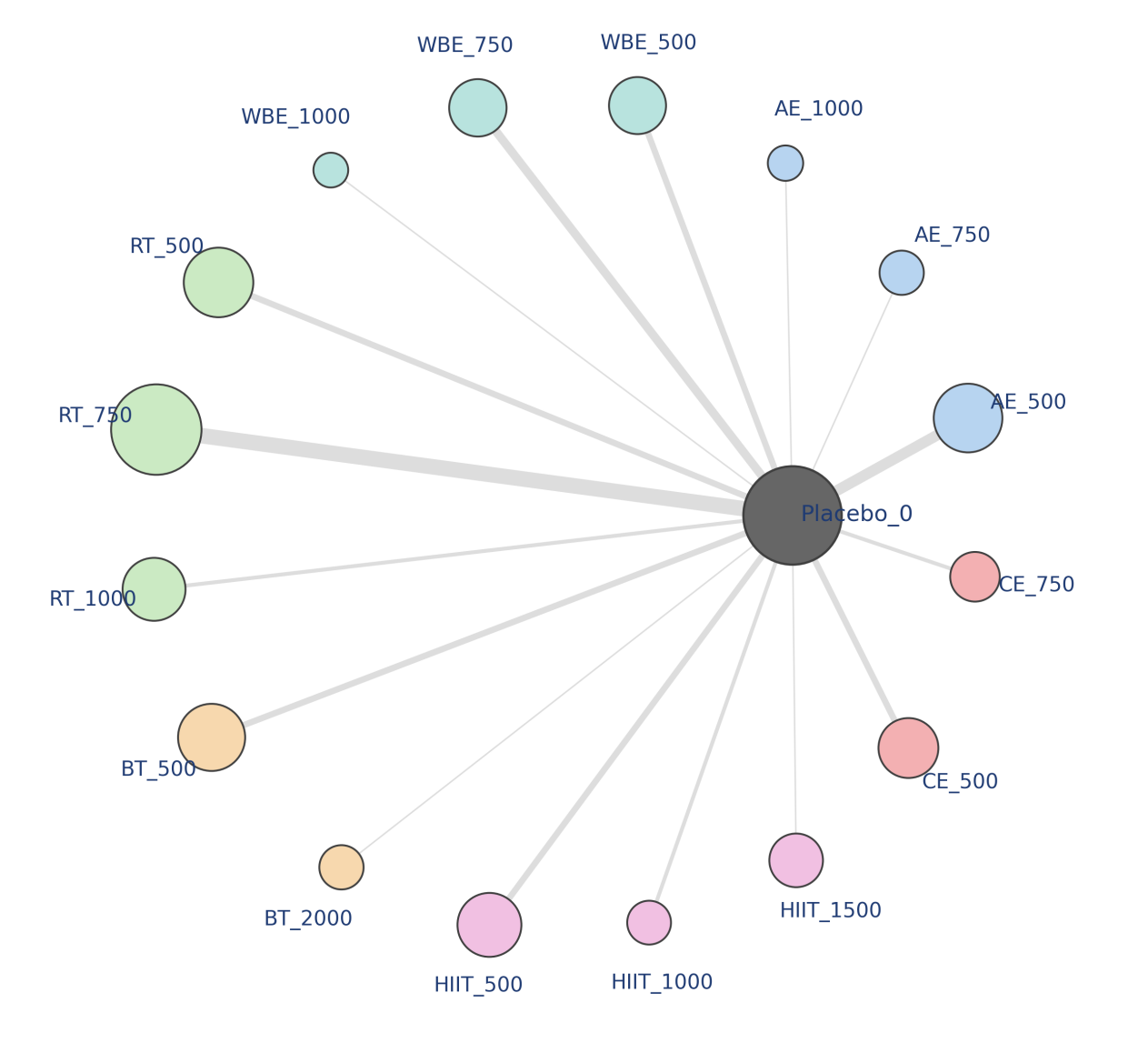


**Supplementary Figure 1**. Treatment-level network.

***Consistency***

We carried out consistency analysis in the data through the comparison of consistent (i.e., network effect sizes) and unrelated mean effects (UME) models (i.e., pairwise effect sizes) of the network. In practice, we checked whether deviance, the number of estimated parameters in the network, and the Deviance Informative Criterion (DIC) indicators were similar for both models which would indicate a good fi. Comparison of these parameters indicated good consistency across models (Supplementary Table 1).

**Supplementary Table 1.** Consistent and UME models fit comparison

| **Model** | **pD** | **Residual deviance** | **DIC** | **SD** |
| --- | --- | --- | --- | --- |
| **Consistent** | **16** | **45.08** | **61.08** | **0.26** |
| **UME** | **16** | **45.08** | **61.08** | **0.26** |

*Note.* pD: Number of estimated parameters; DIC: Deviance Informative Criterion; SD: Standard

Deviation; UME: Unrelated Mean Effects. Scientific literature indicated that the main indicator to

assess the model fit is the DIC. As lower DIC, better fit.

***Transitivity***

NMAs rest under the assumption of underlying indirect/mixed comparisons, which means the estimates of treatment effects from direct and indirect evidence are in agreement, subject to the usual variation under the random-effects model for meta-analysis This assumption is equivalent to heterogeneity in ‘standard’ meta-analysis. Following previous recommendations, transitivity was assessed at the deeper level of the network (i.e., treatment level). We assessed transitivity *via* MBNMA node-splitting approach. This method splits and compares contributions for a particular treatment contrast into direct and indirect evidence. Similar effects denote good transitivity. Supplementary Figures 3 (point estimates) and 4 (density plots) below present the results for transitivity in this meta-analysis.


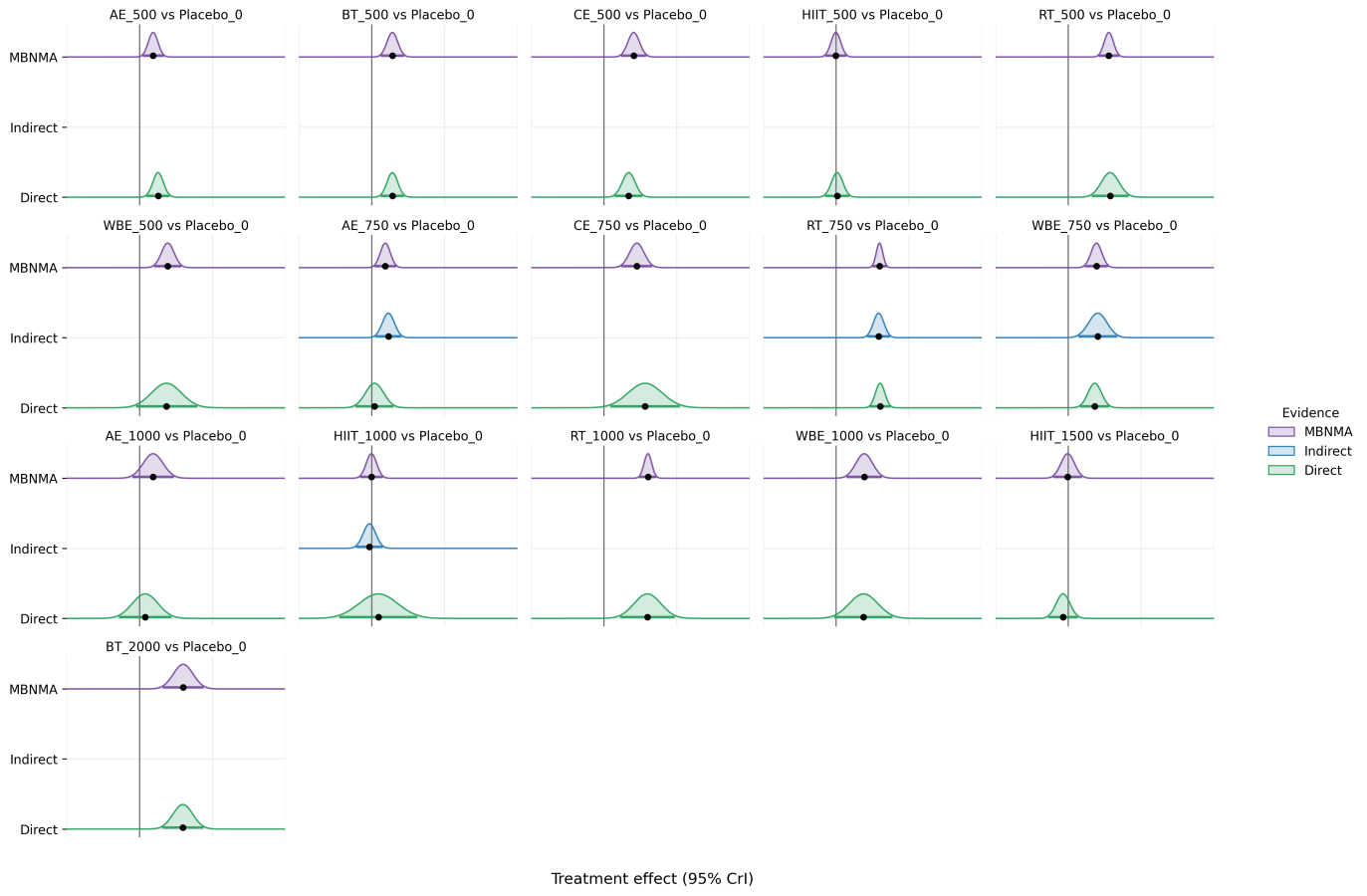


### **Supplementary Figure 2.** Node-splitting analysis (forest plot).


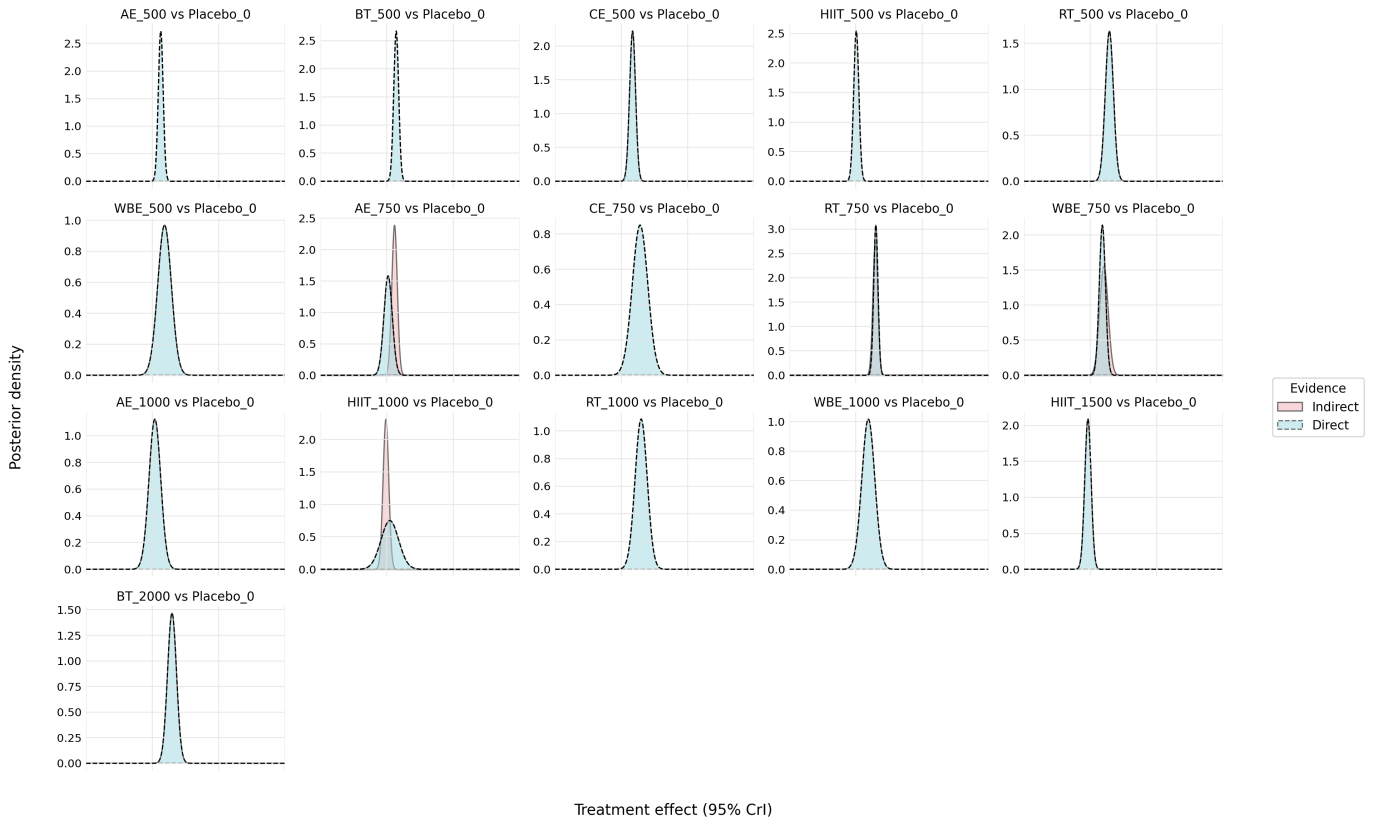


**Supplementary Figure 3.** Node-splitting analysis (density plot).

**Supplementary file 3: Non-linear functions and models fit comparison**

The different doses of physical activity were meta-analysed as independent and unrelated treatments (i.e., “split” NMA). This step is useful to determine which function fits the data better and should subsequently be used in a Model-Based Network Meta-Analysis (MBNMA）. Supplementary Figure 5 and Supplementary Figure 6 show the different responses (Hedges’ g) of each dose for overall and different types of exercise, respectively.


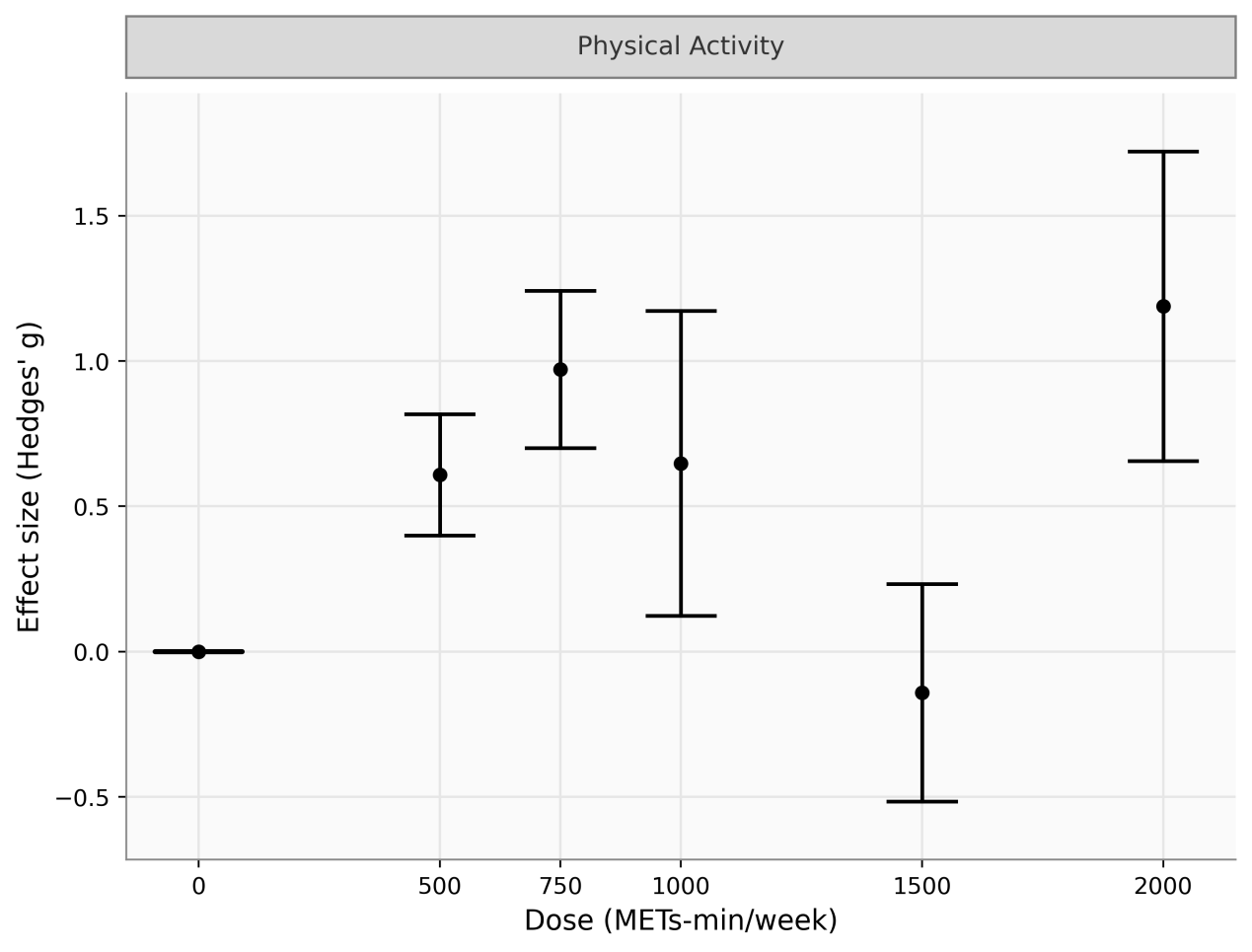
 **Supplementary Figure 4.** “Split” NMA of overall exercise


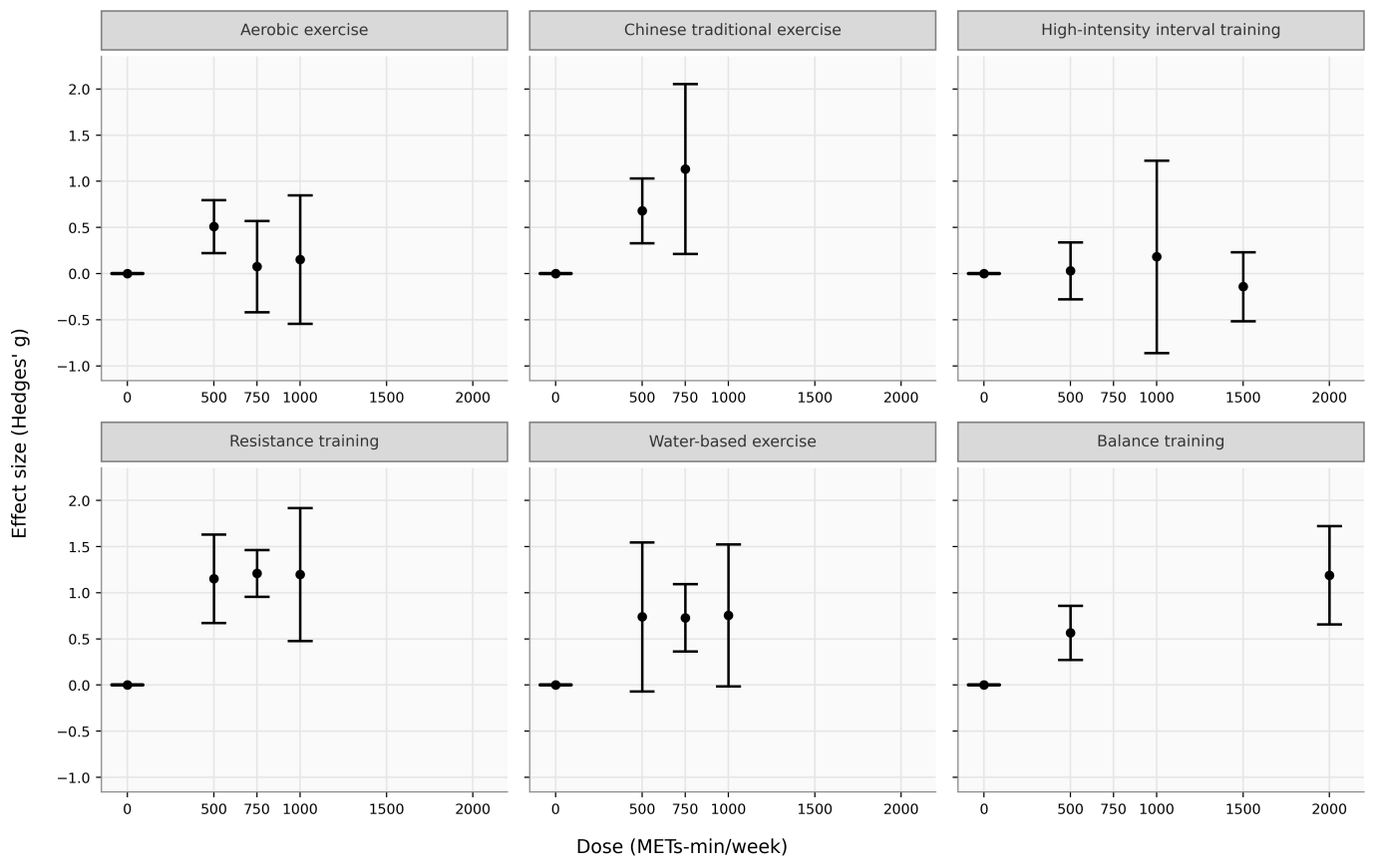


**Supplementary Figure 5.** “Split” NMA of different exercise agents.

Supplementary Table 2 shows the fit indices from each of the models fitted. For our data, restricted cubic splines show the best fit and were therefore used in subsequent analyses.

**Supplementary Table 2.** Models fit comparison

| **Model** | **DIC** | **SD** | **Deviance** | **Residual deviance** | **pD** |
| --- | --- | --- | --- | --- | --- |
| Emax (common treatment effects) | 112.37 | NA | 108.37 | 136.68 | 2 |
| Exponential (common treatment effects) | 112.73 | NA | 108.73 | 137.04 | 2 |
| Restricted cubic spline (common treatment effects; 3 knots) | 113.3 | NA | 103.3 | 131.61 | 5 |
| Restricted cubic spline (random treatment effects; 3 knots) | 78.32 | 0.42 (0.00, 0.48) | 66.32 | 42.95 | 6 |
| Restricted cubic spline (random treatment effects; 4 knots) | 90.11 | 0.00 (0.00, 0.42) | 76.11 | 104.42 | 7 |
| Non-parametric monotonically up (common treatment effects) | 112.73 | NA | 100.73 | 129.04 | 6 |

*Note.* DIC = Deviance Information Criterion; SD = Between-study Standard Deviation; pD: Number of estimated parameters; NA = Not Applicable. The SD is presented as the main value and (95% Credible Intervals).

Further to model fit indices, deviance plots showing the contribution of each data point to the residual deviance are also useful to confirm the robustness of model selection[[9]](https://paperpile.com/c/b8T1z2/q25h). Each data point should contribute about 1 to the posterior mean deviance, which indicates good model fit[[10]](https://paperpile.com/c/b8T1z2/6GsS). The deviance plot for overall (Supplementary File 7) and treatment effects (Supplementary File 8) confirm the robustness of our model selection (i.e., deviances < 1.5 except for few data points at 500 METs-min in overall exercise and resistance bands, all below a contribution of 2.5).


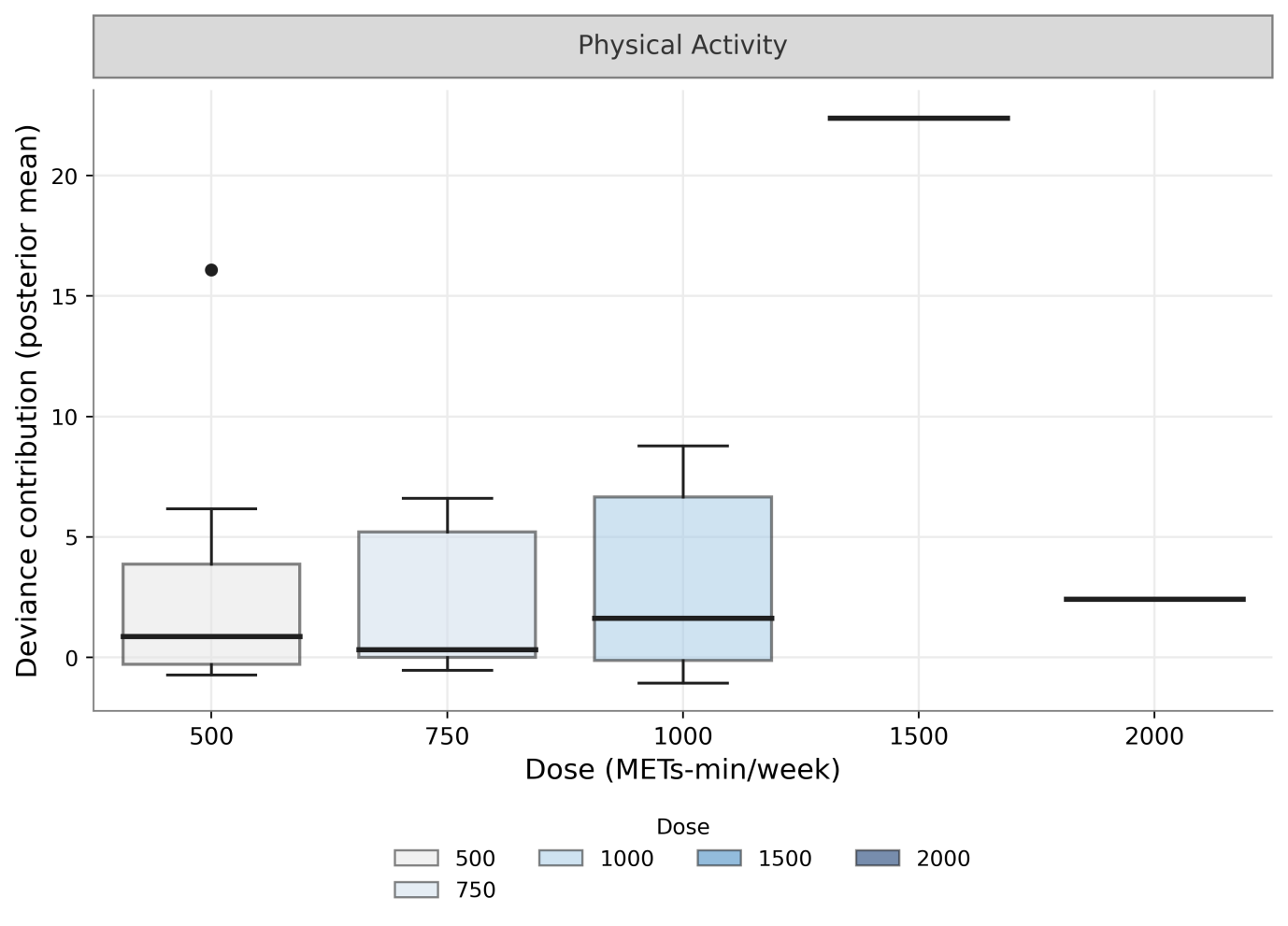


**Supplementary Figure 6**. Deviance plot at overall exercise level.


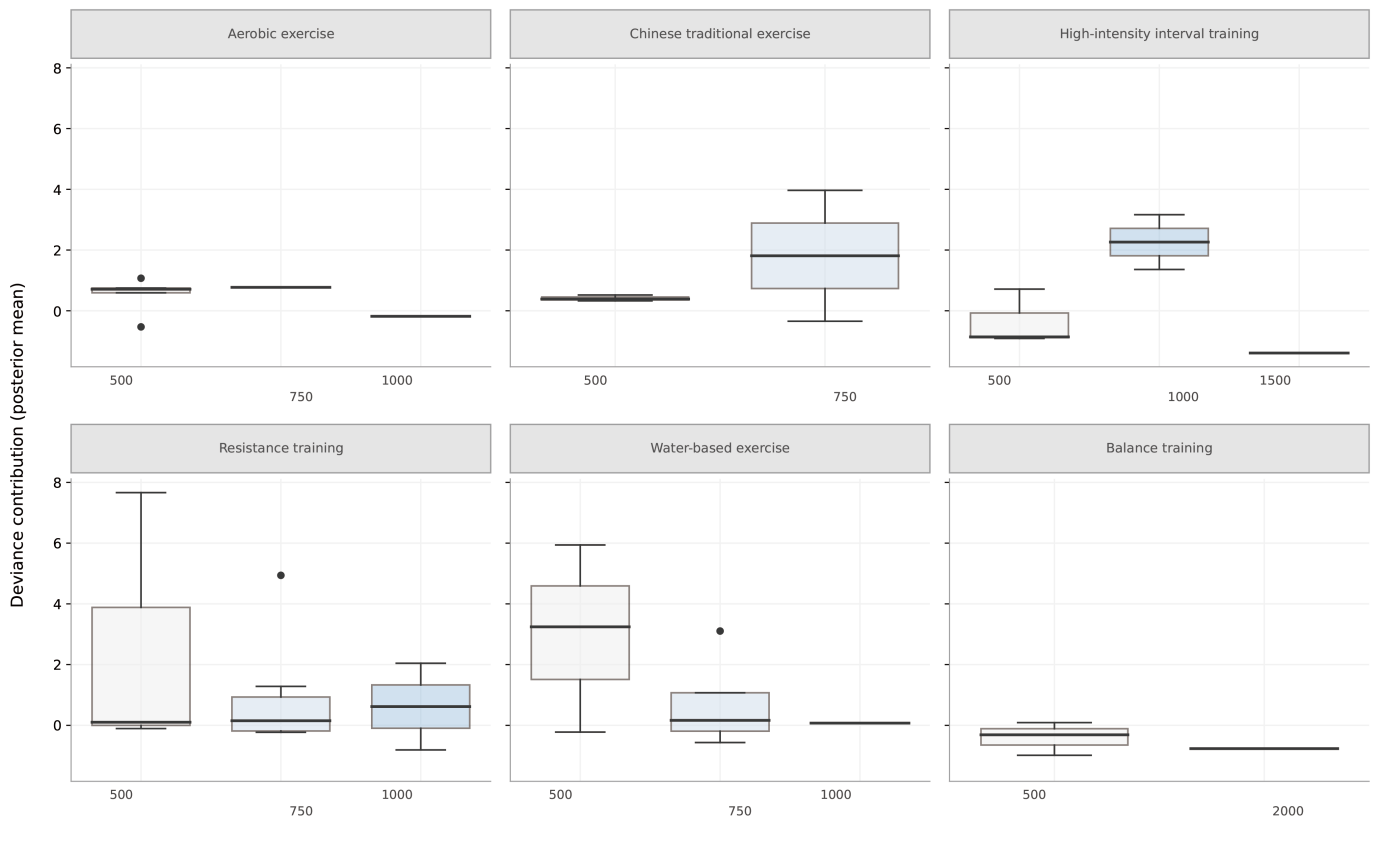


**Supplementary Figure 7.** Deviance plots at treatment-level.

**Supplementary file 4: Characteristics of included studies**

| **Study** | **Age (E/C)** | **Sample size n (M/F) (E/C)** | **Exercise type** | **Session duration (min/session)** | **Frequency (sessions/week)** | **Intervention period (weeks)** | **Dose_week (MET-min/week)** | **Dose_group (mapped)** | **Dose_day (MET-min/day)** | **Outcome measure(s)** |
| --- | --- | --- | --- | --- | --- | --- | --- | --- | --- | --- |
| Li et al. (2014)[1] | E: 73.32±7.58 C: 73.39±8.58 | E: 28 (14/14) C: 28 (14/14) | Aerobic exercise | 60 | 3 | 16 | **540** | **500** | **77.142857** | BBS |
| Globas et al. (2012)[2] | E: 68.6±6.7 C: 68.7±6.1 | E: 18 (14/4) C: 18 (13/5) | Aerobic exercise | 30-50 | 3 | 12 | 420 | 500 | 60 | BBS |
| Moore et al. (2015)[3] | E: 68±8 C: 70±11 | E: 20 (18/2)  C: 20 (16/4) | Aerobic exercise | 45-60 | 3 | 19 | 551.25 | 500 | 78.75 | BBS |
| Outermans et al. (2010)[4] | E: 56.8±8.6  C: 56.3±8.6 | E: 22 (19/3)  C: 21 (17/4) | Aerobic exercise | 45 | 3 | 4 | 472.5 | 500 | 67.5 | BBS |
| Hornby et al. (2016)[5] | E: 57±12; C: 60±9.2 | E: 15 (12/3) 、C: 17 (12/5) | Aerobic exercise | 60 | 4-5 | 10 | 945 | 1000 | 135 | BBS |
| Kim et al. (2015)[6] | E: 52.5±11.72  C: 53.4±12.11 | E: 10 C: 10 | Aerobic exercise | 60 | 3 | 4 | 540 | 500 | 77.142857 | BBS |
| Pang et al. (2005)[7] | E: 65.8±9.1  C: 64.7±8.4 | E: 32 (19/13) C: 31 (18/13) | Aerobic exercise | 60 | 3 | 19 | 630 | 750 | 90 | BBS |
| Saeys et al. (2012)[8] | E: 61.94±13.83  C: 61.07±9.01 | E: 18 (9/9) C: 15 (8/7) | Resistance exercise | 30 | 4 | 8 | 420 | 500 | 60 | BBS |
| Li et al. (2019)[9] | E: 55.43±8.12  C: 54.30±9.32 | E: 25 (13/12) C: 24 (14/10) | Resistance exercise | 30-50 | 5 | 8 | 700 | 750 | 100 | BBS |
| Li et al. (2013)[10] | E: 48.4±2.3  C: 50.2±8.4 | E: 26 (14/12) C: 24 (11/13) | Resistance exercise | 20 | 10 | 6 | 700 | 750 | 100 | BBS |
| Zhang et al. (2014)[11] | E: 65.13±5.38  C: 62.36±6.43 | E: 20 (12/8) C: 20 (13/7) | Resistance exercise | 40 | 5 | 6 | 700 | 750 | 100 | BBS |
| Li et al. (2015)[12] | E: 59.10±12.56  C: 58.30±16.47 | E: 30 (19/11) C: 30 (13/17) | Resistance exercise | 40 | 6 | 8 | 840 | 750 | 120 | BBS |
| Xi et al. (2011)[13] | E: 63.1±12.2  C: 61.8±11.6 | E: 20 (12/8) C: 20 (13/7) | Resistance exercise | 40 | 5 | 6 | 700 | 750 | 100 | BBS |
| Fu et al. (2016a)[14] | E: 59.7±7.6  C: 60.3±8.4 | E: 30 (19/11) C: 30 (18/12) | Resistance exercise | 40 | 6 | 6 | NR | NR | NR | BBS |
| Shen et al. (2013)[15] | E:59.23±12.85;  C: 58.18±13.16 | E: 40 (21/19) C: 40 (23/17) | Resistance exercise | 30 | 10 | 4 | 1050 | 1000 | 150 | BBS |
| Liu et al. (2014)[16] | E: 63.7±10.9  C: 63.2±9.9 | E: 53 (27/26) C: 52 (31/21) | Resistance exercise | 15 | 5 | 4 | 262.5 | 500 | 37.5 | BBS |
| Zeng et al. (2016)[17] | E: 56.5±6.2  C: 54.8±5.6 | E: 40 (21/19) C: 40 (22/18) | Resistance exercise | 45 | 6 | 6 | 945 | 1000 | 135 | BBS |
| Cabanas-Valdés et al. (2016)[18] | E: 74.92±10.70  C: 75.69±9.40 | E: 40 (21/19) C: 39 (18/21) | Resistance exercise | 75 | 5 | 5 | 1312.5 | 1500 | 187.5 | BBS |
| He et al. (2012)[19] | E: 57.81±11.22  C: 55.92±12.75 | E: 30 (19/11) C: 30 (17/13) | Resistance exercise | 15 | 6 | 4 | 315 | 500 | 45 | BBS |
| Chu et al. (2004)[20] | E: 61.9±9.4  C: 63.4±8.4 | E: 7 (6/1) C: 5 (5/0) | Aquatic exercise | 60 | 3 | 8 | 720 | 750 | 102.8571429 | BBS |
| Ku et al. (2020)[21] | E: 55±7.3  C: 52.5±6.3 | E: 10 (7/3) C: 10 (7/3) | Aquatic exercise | 60 | 3 | 6 | 720 | 750 | 102.8571429 | BBS |
| Tripp et al. (2014)[22] | E: 64.8±15.0  C: 65.0±15.1 | E: 14 (9/5) C: 16 (10/6) | Aquatic exercise | 45 | 3 | 2 | 540 | 500 | 77.14285714 | BBS |
| Zhu et al. (2016)[23] | E: 56.6±6.9  C: 57.1±8.6 | E: 14 (12/2) C: 14 (10/4) | Aquatic exercise | 45 | 5 | 4 | 900 | 1000 | 128.5714286 | BBS |
| Lee et al. (2018)[24] | E: 57.58±13.98  C: 63.67±11.37 | E: 19 (9/10) C: 18 (10/8) | Aquatic exercise | 30 | 5 | 4 | 600 | 500 | 85.71428571 | BBS |
| Eyvaz et al. (2018)[25] | E: 58.5±6.27  C: 58.3±5.43 | E: 30 (12/18) C: 30 (17/13) | Aquatic exercise | 60 | 3 | 6 | 720 | 750 | 102.8571429 | BBS |
| Zeng Ming et al. (2019)[26] | E: 63.18±9.47  C: 61.10±12.32 | E: 17 (9/8) C: 16 (10/6) | Aquatic exercise | 45 | 5 | 6 | 787.5 | 750 | 112.5 | BBS |
| Wang Li et al. (2014)[27] | E: 48.56±5.26  C: 51.65±4.91 | E: 30 (22/8) C: 30 (20/10) | Aquatic exercise | 30 | 5 | 6 | 525 | 500 | 75 | BBS |
| Xie Rongqing et al. (2019)[28] | E: 51.10±12.92  C: 53.95±13.00 | E: 20 (13/7) C: 20 (12/8) | Traditional Chinese exercise | 50 | 5 | 3 | 750 | 750 | 107.1428571 | BBS |
| Fu et al. (2016b）[29] | E: 59.7±7.6  C: 60.3±8.4 | E: 30 (19/11) C: 30 (18/12) | Traditional Chinese exercise | 40 | 6 | 8 | 720 | 750 | 102.8571429 | BBS |
| Liu et al. (2009)[30] | E: 52.13±14.13  C: 53.51±12.63 | E: 24 (14/10) C: 24 (11/13) | Traditional Chinese exercise | 30 | 7 | 12 | 630 | 750 | 90 | BBS |
| Wang et al. (2016)[31] | E: 60.71±7.32  C: 58.56±8.52 | E: 14 (9/5) C: 16 (14/2) | Traditional Chinese exercise | 60 | 5 | 12 | 600 | 500 | 85.71428571 | BBS |
| Yang et al. (2016)[32] | E: 51.43±15.63  C: 54.85±11.85 | E: 28 (17/9) C: 21 (14/7) | Traditional Chinese exercise | 40 | 5 | 8 | 600 | 500 | 85.71428571 | BBS |
| He et al. (2022)[33] | E: 62.96±8.98  C: 62.50±10.73 | E: 26 (20/6) C: 29 (23/6) | Traditional Chinese exercise | 30 | 6 | 4 | 540 | 500 | 77.14285714 | BBS |
| Büyükavcı et al. (2016)[34] | Not reported | E: 32 C: 32 | Resistance exercise | 120 | 7 | 3 | 2520 | 2000 | 360 | BBS |
| Ordahan et al. (2015)[35] | Not reported | E: 25 C: 25 | Resistance exercise | 20 | 5 | 6 | 300 | 500 | 42.85714286 | BBS |
| Yoo et al. (2010)[36] | Not reported | E: 28 C: 31 | Resistance exercise | 30 | 3 | 4 | 270 | 500 | 38.57142857 | BBS |
| Globas et al. (2012)[37] | E: 68.6±6.7 C: 68.7±6.1 | E: 18 C: 18 | High intensity training | 30–50 | 3 | 8 | 900 | 1000 | 128.5714286 | BBS |
| Mberti et al. (2017)[38] | E: 67±10 C: 69±9 | E: 17 C: 18 | High intensity training | 30 | 3 | 8 | 360 | 500 | 51.42857143 | BBS |
| Lau et al. (2011)[39] | E: 69.5±11.1 C: 72.1±9.2 | E: 15 C: 15 | High intensity training | 30 | 3 | 4 | 720 | 1000 | 102.8571429 | BBS |
| Moore et al. (2020)[40] | E: 73±10 C: 74±14 | E: 54 C: 56 | High intensity training | 45–60 | 4 | 3 | 1300 | 1500 | 185.7142857 | BBS |
| Pang et al. (2005)[41] | E: 65.8±9.1 C: 64.7±8.4 | E: 32 C: 31 | High intensity training | 30 | 3 | 19 | 540 | 500 | 77.14285714 | BBS |
| Gjellesvik et al. (2021)[42] | E: 57.6±9.2 C: 58.7±9.2 | E: 36 C: 34 | High intensity training | 17 | 3 | 8 | 400 | 500 | 57.14285714 | BBS |

eg:BBS: Berg Balance Scale

**Supplementary file 5: Study-level Risk of Bias analysis**

Supplementary Figure 8 shows the risk of bias for each included study and domain analyzed according to the Cochrane Risk of Bias Tool.

**
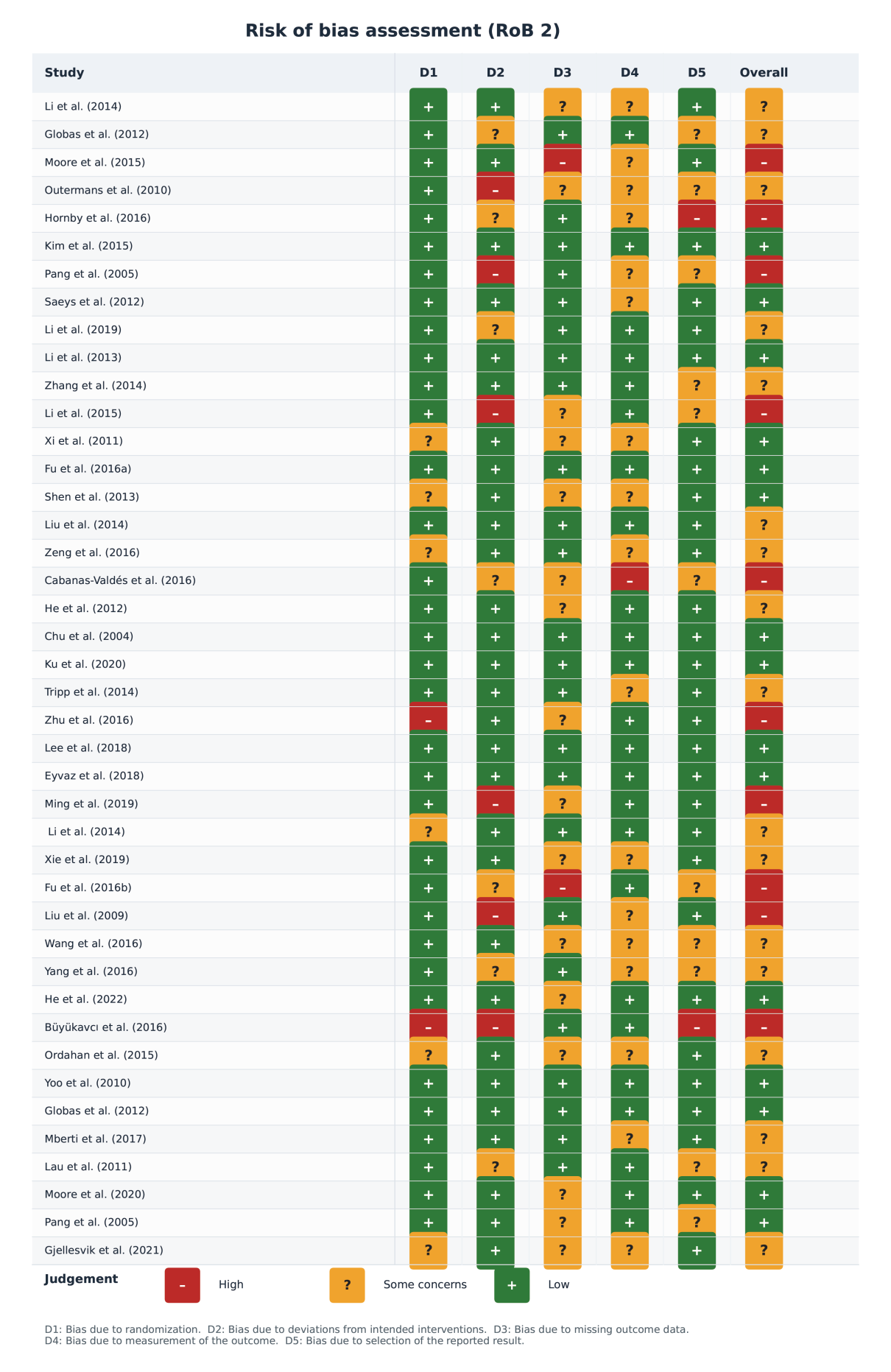
**

**Supplementary Figure 8.The detail of risk of bias**

Supplementary File9 provides the detailed CINeMA (Confidence in Network Meta-Analysis) assessments for all estimable comparisons in the network (Figure Sx). Each row corresponds to one comparison (e.g., AE vs BT), and columns D1–D6 represent the six CINeMA domains: D1 within-study bias, D2 reporting bias, D3 indirectness, D4 imprecision, D5 heterogeneity, and D6 incoherence. Colour coding indicates the level of concern for each domain (no concerns, some concerns, major concerns), and the rightmost column reports the overall confidence rating (high, moderate, low, or very low) for each comparison.


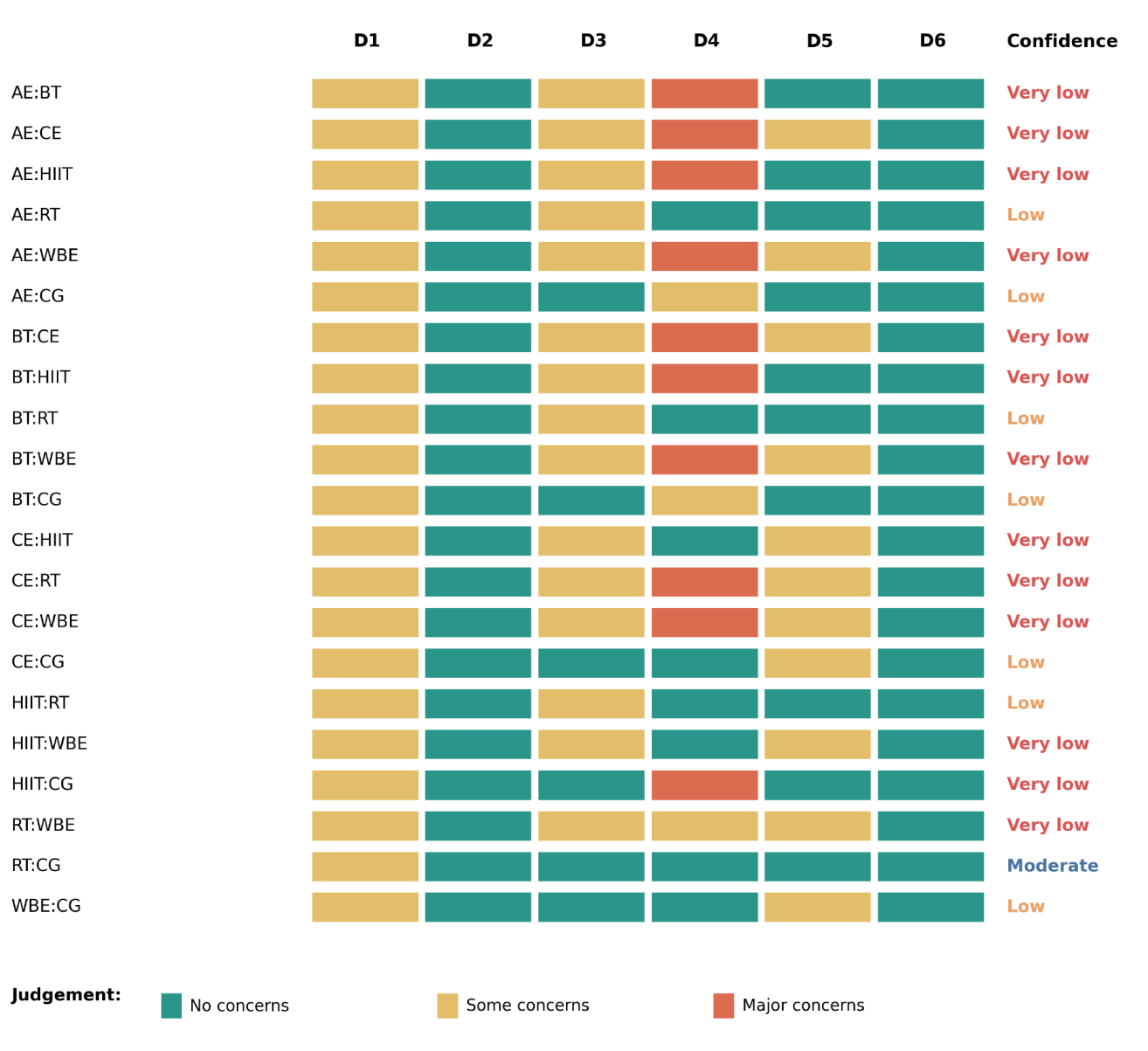


**Supplementary Figure 9.The detail of CINeMA Confidence**

**Supplementary file 6: Predicted responses at 600, 1200, and 1800 METs per week**

Supplementary Table 4 shows the predicted effects for the lower and upper bound of WHO recommended level of physical activity as well as corresponding effects for the minimum WHO recommended level of physical activity for each type of intervention considered in our analysis.

| Supplementary Table 4. Predicted responses at 600, 1200, and 1800 METs-min per week | | | | |
| --- | --- | --- | --- | --- |
| Agent | Exercise Dose (METs-min/week) | Mean | SD | 95% CrI |
| Aerobic exercise | 600 | 0.47 | 0.32 | (0.16, 1.42) |
| Aerobic exercise | 1200 | -0.15 | 3.34 | (-4.73, 9.78) |
| Aerobic exercise | 1800 | -0.82 | 6.41 | (-9.79, 18.14) |
| Resistance training | 600 | 1.33 | 0.22 | (0.92, 1.76) |
| Resistance training | 1200 | 0.69 | 0.49 | (-0.19, 1.83) |
| Resistance training | 1800 | -0.39 | 1.28 | (-2.63, 2.63) |
| Chinese traditional exercise | 600 | 0.92 | 0.21 | (0.59, 1.44) |
| Chinese traditional exercise | 1200 | 3.63 | 7.75 | (-3.38, 19.79) |
| Chinese traditional exercise | 1800 | 6.21 | 15.7 | (-8.78, 38.92) |
| Water-based exercise | 600 | 0.77 | 0.26 | (0.25, 1.28) |
| Water-based exercise | 1200 | 0.12 | 1.21 | (-2.80, 2.11) |
| Water-based exercise | 1800 | -0.75 | 2.86 | (-7.35, 4.14) |
| Balance training | 600 | 1.94 | 2.09 | (-2.31, 5.63) |
| Balance training | 1200 | 4.32 | 6.46 | (-8.62, 16.03) |
| Balance training | 1800 | 6.3 | 10.99 | (-14.92, 26.42) |
| High-intensity interval training | 600 | 0.07 | 0.19 | (-0.26, 0.42) |
| High-intensity interval training | 1200 | 0.2 | 0.81 | (-1.50, 1.92) |
| High-intensity interval training | 1800 | 0.26 | 1.78 | (-2.94, 4.47) |

*Predicted responses (SMD, Hedges’ g) and 95% Credible Intervals (95% CrI). Bold represent effects for which corresponding 95% Credible Intervals do not include zero. SD = Standard Deviation.*

**Supplementary File 7: Ranking of effectiveness of interventions**

Supplementary Figure 10 shows the probability of each intervention to be ranked from worst to best (estimated after up to 4000 iterations).

***
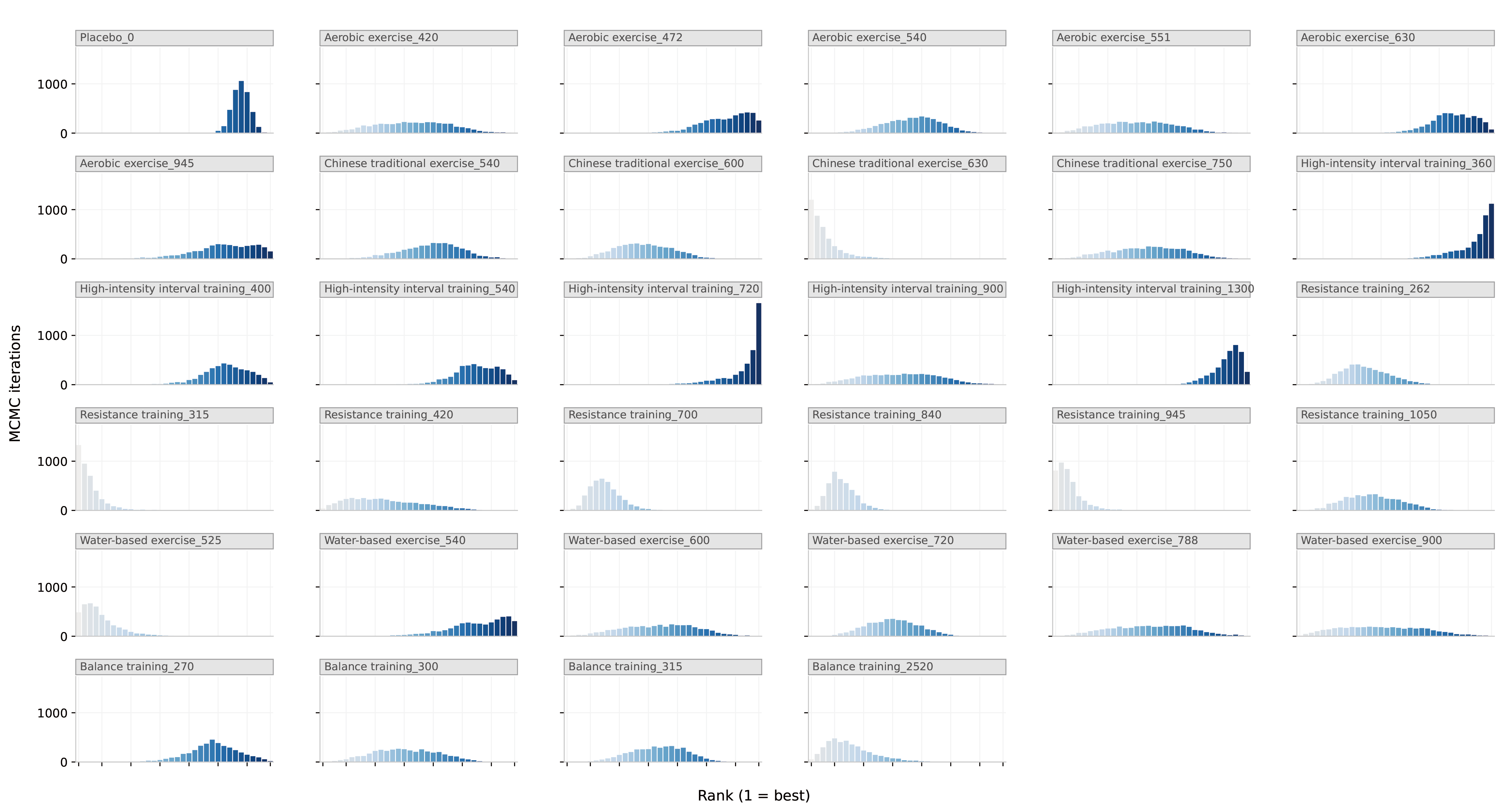
***

**Supplementary Figure 10.** Effectiveness ranking by exercise treatments. The number that follows the exercise intervention indicates the dose of exercise (METs-min/week)

**Supplementary File 8. Sensitivity analyses**

This supplement reports sensitivity analyses to assess robustness of the main conclusions.

**S8.2 Risk of bias sensitivity analyses**

Aim: Evaluate robustness of modality-level pooled effects and overall dose–response conclusions after excluding trials rated as overall high risk of bias (RoB 2).

**Excluded trials (overall high risk):** Büyükavcı, et all. 2016; Cabanas, et all.2016; Fu, et all. (2016)b; Hornb , et all. (2015); Li, et all. (2015); Liu, et all. (2009); Moore, et all. (2014); Pang, et all. 2005; Pang, et all.(2005); Zeng, et all.(2019); Zhu , et all. (2016).

**S8.2.1 Modality-level pooled effects versus control**

We re-estimated pooled effects for each modality using a Bayesian random-effects meta-analysis on the standardized mean difference scale (Hedges’ g), first using all included trials and then excluding trials rated as overall high risk of bias. Results are presented as posterior means with 95% credible intervals (CrIs).

| **Modality** | **k (Main)** | **Main (mean g)** | **Main 95% CrI** | **k (Excl high RoB)** | **Excl high RoB (mean g)** | **Excl high RoB 95% CrI** |
| --- | --- | --- | --- | --- | --- | --- |
| RT | 12 | 1.19 | (0.99, 1.39) | 10 | 1.15 | (0.75, 1.56) |
| CE | 5 | 0.88 | (0.48, 1.28) | 4 | 0.69 | (-0.22, 1.55) |
| BT | 4 | 0.72 | (0.35, 1.08) | 2 | 0.44 | (-1.17, 1.92) |
| WBE | 8 | 0.72 | (0.39, 1.06) | 6 | 0.64 | (-0.09, 1.35) |
| AE | 7 | 0.38 | (0.13, 0.62) | 4 | 0.68 | (-0.27, 1.59) |
| HIIT | 6 | 0.02 | (-0.24, 0.27) | 5 | 0.01 | (-0.77, 0.76) |

Interpretation: Excluding overall high-risk trials did not materially change the direction of effects for RT and HIIT; however, uncertainty increased for modalities with few remaining trials (e.g., BT), as reflected by wider CrIs.

**S8.2.2 Overall dose–response robustness**

We refit the overall random-effects spline dose–response model after excluding overall high-risk trials. Convergence diagnostics for both models are provided in Supplementary File 9. Key summaries are reported below.

Main model heterogeneity (tau median, 95% CrI): 0.527 (0.404–0.701); Excluding high RoB: 0.534 (0.397–0.740).

Main model diagnostics: max R-hat=1.000097, min bulk ESS=12028, min tail ESS=11044; Excluding high RoB: max R-hat=1.000214, min bulk ESS=12124, min tail ESS=11259.

Across the observed dose range, the qualitative non-linear pattern was broadly consistent, but uncertainty increased after excluding high-risk trials. As a result, point estimates of the peak region were less stable and should be interpreted as hypothesis-generating rather than definitive.

**Comparison of SMD- and MD-based pooled estimates by modality**

This supplementary comparison was added to improve clinical interpretability under a common outcome scale (BBS). Hedges’ g was retained as the primary effect-size metric for the main Bayesian network and dose–response analyses, whereas mean difference (MD) on the original BBS scale is shown here as a modality-level supplementary comparison.

| **Modality** | **k** | **Primary analysis: Hedges' g** | **95% CrI** | **Supplementary comparison: MD** | **95% CrI** | **Direction consistent?** |
| --- | --- | --- | --- | --- | --- | --- |
| Resistance training | 12 | 1.19 | (0.99, 1.39) | 6.01 | (4.33, 7.92) | Yes |
| Chinese exercise | 5 | 0.88 | (0.48, 1.28) | 4.99 | (1.14, 8.96) | Yes |
| Balance training | 4 | 0.72 | (0.35, 1.08) | 7.62 | (3.03, 12.18) | Yes |
| Water-based exercise | 8 | 0.72 | (0.39, 1.06) | 4.72 | (2.50, 6.83) | Yes |
| Aerobic exercise | 7 | 0.38 | (0.13, 0.62) | 2.01 | (0.27, 4.11) | Yes |
| HIIT | 6 | 0.02 | (-0.24, 0.27) | 0.02 | (-1.58, 1.77) | Yes |

Interpretation: The direction of pooled effects was consistent between the primary Hedges’ g analysis and the supplementary MD-based comparison across all modalities.

**Supplementary File 9. Bayesian model transparency and MCMC diagnostics**

This supplementary file provides a concise summary of the Bayesian computational workflow used to support transparency and diagnostic assessment of the analyses reported in the manuscript. It reports the software environment, MCMC settings, quantitative convergence diagnostics, and representative diagnostic plots.

Important clarification. The primary analyses reported in the main manuscript were conducted in R, as described in the main Methods. The Python-based implementation documented in this supplementary file was used only as an additional supplementary verification and diagnostic workflow. It was included to reproduce key posterior summaries and to present convergence diagnostics more transparently; it does not replace the primary R-based analysis pipeline.

**Outcome and effect-size framework**

The primary outcome was the Berg Balance Scale (BBS). For the primary Bayesian analyses, trial-level contrasts were summarized on the standardized mean difference scale (Hedges’ g), and corresponding standard errors were used as model inputs.

**Model overview**

Two Bayesian model components were evaluated in this supplementary verification workflow: (1) modality-level random-effects synthesis, using a common between-study heterogeneity parameter (τ); and (2) a dose–response verification model, in which dose was treated as continuous MET-min/week and the overall non-linear dose–response relationship was represented using restricted cubic splines. Detailed mathematical derivations are not repeated here because the purpose of this supplementary file is diagnostic transparency rather than full methodological redevelopment.

**Priors**

Weakly informative priors were used throughout the supplementary Bayesian verification analysis. Treatment effects and spline coefficients were assigned normal priors, and between-study heterogeneity was assigned a weakly informative half-Student-t prior.

**Computation and MCMC settings**

As a supplementary verification step, we implemented a JAGS-consistent conjugate Gibbs sampler in Python. The analysis used 4 independent chains, 50,000 iterations per chain, 20,000 burn-in iterations, thinning = 10, and 12,000 retained posterior draws in total. Because this workflow used a Gibbs sampler, sampler settings such as adapt_delta or max_treedepth were not applicable.

**Convergence diagnostics**

Convergence and mixing were evaluated using R-hat, effective sample size (ESS; bulk and tail), and representative trace plots and posterior density plots. A summary of the quantitative convergence diagnostics is provided in Table S9. Representative graphical diagnostics are shown in Supplementary Figure S11, which includes trace plots and posterior density plots for the heterogeneity parameter (τ) and selected spline coefficients from the dose–response model.

**Summary of diagnostic results**

Quantitative diagnostics indicated satisfactory convergence and mixing across monitored parameters: max R-hat = 1.000097; min bulk ESS = 12,028; min tail ESS = 11,044. Between-study heterogeneity was moderate: tau median = 0.527, 95% CrI = 0.404–0.701. These results support adequate convergence of the supplementary Bayesian verification workflow.

**Interpretation**

The credible intervals reported in the main text describe uncertainty in the estimated mean effects conditional on the fitted model. They should not be interpreted as equivalent to study-level predictive uncertainty. Between-study variability is better reflected by τ and, where relevant, predictive intervals.

**Supplementary Table S9. Convergence diagnostics for Bayesian models**

| **Model** | **Parameters monitored** | **Max R-hat** | **Min ESS (bulk)** | **Chains / iter / warmup** | **Notes** |
| --- | --- | --- | --- | --- | --- |
| NMA (modality-level) | d_t; τ | 1.000097 | 12,028 | 4 / 50,000 / 20,000 (thin=10) | tau median 0.527 (95% CrI 0.404–0.701) |
| MBNMA (dose–response) | spline coeffs; τ | 1.000097 | 12,028 | 4 / 50,000 / 20,000 (thin=10) | tau median 0.527 (95% CrI 0.404–0.701); min tail ESS 11,044 |

For communication anchors, model-estimated mean effects (median, 95% CrI) at 600/1200/1800 MET-min/week were 0.736 (0.120–1.364), 0.798 (0.519–1.071), and 0.611 (−0.491–1.729), respectively; corresponding predictive distributions for a new study effect (median, 95% predictive interval) were 0.737 (−0.496–1.989), 0.802 (−0.299–1.893), and 0.616 (−0.923–2.166).

**Supplementary Figure S11. Trace plots and posterior density for representative monitored parameters**

Representative monitored parameters from the supplementary Bayesian verification analysis are shown, including three spline coefficients from the dose–response model and the heterogeneity parameter (τ), across four independent chains. The left panels show trace plots and the right panels show posterior density distributions by chain. These displays were inspected together with the quantitative diagnostics in Table S9 to assess chain mixing, stability, and convergence.


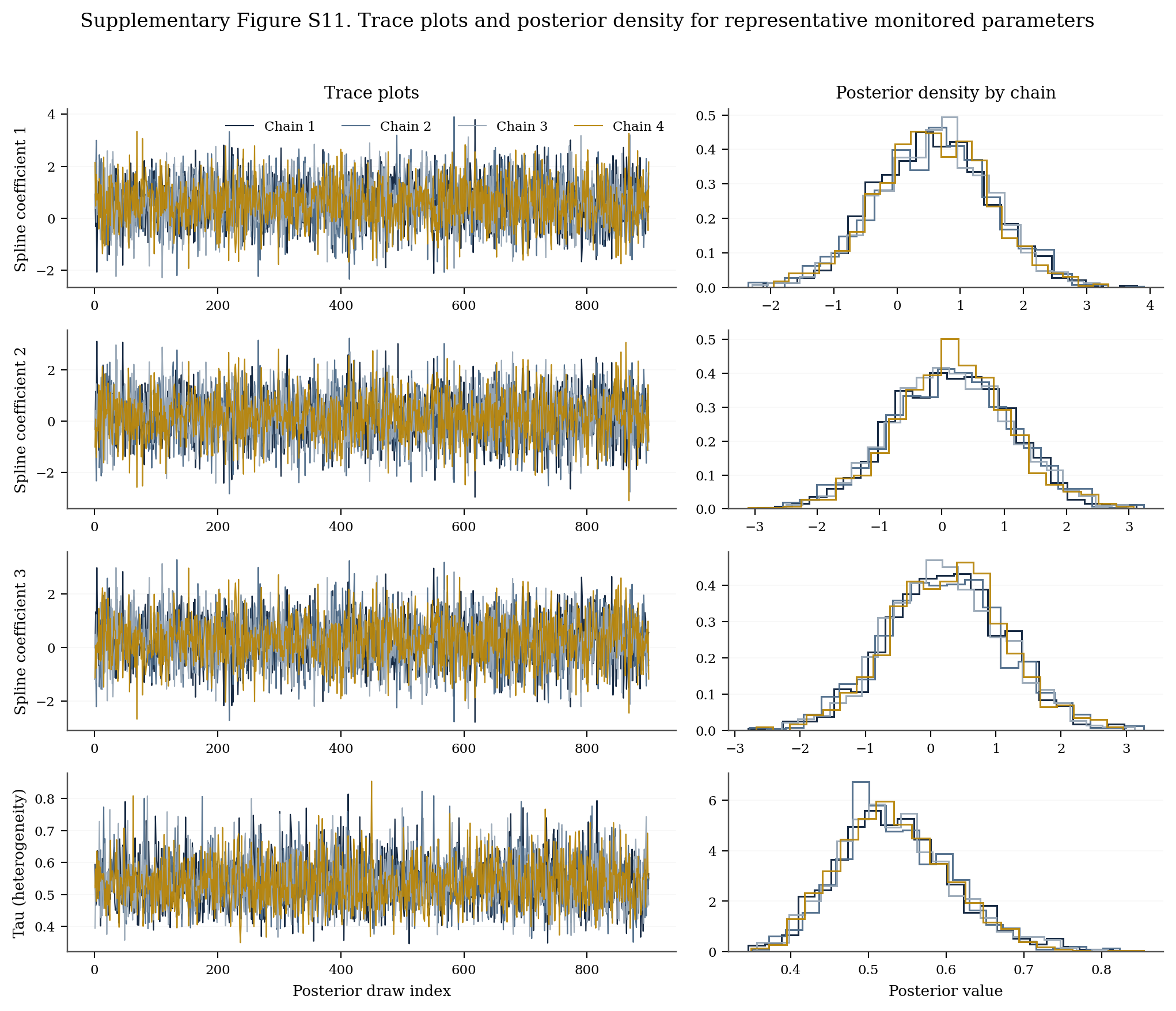


Abbreviation: τ, between-study heterogeneity standard deviation.

**References**

[1] Li Y, Cheng Y, Zhao LR, et al. Effect of the Otago Exercise Program on fear of falling in older stroke patients [In Chinese]. Chinese Journal of Nursing. 2014;49(12):1442-1447.

[2] Globas C, Becker C, Cerny J, et al. Chronic stroke survivors benefit from high-intensity aerobic treadmill exercise: A randomized control trial. Neurorehabilitation and Neural Repair. 2012;26(1):85-95.

[3] Moore SA, Hallsworth K, Jakovljevic DG, et al. Effects of community exercise therapy on metabolic, brain, physical, and cognitive function following stroke: A randomized controlled pilot trial. Neurorehabilitation and Neural Repair. 2015;29(7):623-635.

[4] Outermans JC, van Peppen RPS, Wittink H, et al. Effects of a high-intensity task-oriented training on gait performance early after stroke: A pilot study. Clinical Rehabilitation. 2010;24(11):979-987.

[5] Hornby AT, Holleran CL, Hennessy PW, et al. Variable intensive early walking poststroke (VIEWS): A randomized controlled trial. Neurorehabilitation and Neural Repair. 2016;30(5):440-450.

[6] Kim HY, Kim YL, Lee SM. Effects of therapeutic Tai Chi on balance, gait, and quality of life in chronic stroke patients. Int J Rehabil Res. 2015;38(2):156-161.

[7] Pang MY, Eng JJ, Dawson AS, et al. A community-based fitness and mobility exercise program for older adults with chronic stroke: A randomized, controlled trial. J Am Geriatr Soc. 2005;53(10):1667-1674.

[8] Saeys W, Vereeck L, Truijen S, et al. Randomized controlled trial of truncal exercises early after stroke to improve balance and mobility. Neurorehabilitation and Neural Repair. 2012;26(3):231-238.

[9] Li Y, Fu JM, Li H, et al. Effects of four-limb coordinated training on balance and motor function in stroke patients. Chinese Journal of Rehabilitation Medicine. 2019;34(1):78-80.

[10] Li H, Li Y, Gu XD, et al. Effects of intensive trunk muscle training combined with stair training on balance and lower-limb function in stroke patients. Chinese Journal of Physical Medicine and Rehabilitation. 2013;35(5):426-427.

[11] Zhang B, Ding D, Lv L. Effects of proprioceptive training combined with core stability training on lower-limb function and balance in post-stroke hemiplegia patients . Chinese Journal of Rehabilitation Theory and Practice. 2014;20(12):1109-1112.

[12] Li J, Su JJ, Wu Q, et al. Effects of core muscle training on balance function in post-stroke hemiplegia patients . Chinese Journal of Physical Medicine and Rehabilitation. 2015;37(9):678-679.

[13] Xi JM, Feng XD. Effects of core strength training on walking and balance ability in stroke patients. Chinese Journal of Rehabilitation Medicine. 2011;26(8):777-778.

[14] Fu CX, Zhang QY. Effects of core stability training on balance function and walking ability in post-stroke hemiplegia patients [In Chinese]. Chinese Journal of Gerontology. 2016;36(21):5397-5398.

[15] Shen Y, Wang WW, Chen Y, et al. Effects of core stability training on standing balance and walking ability in post-stroke hemiplegia patients. Chinese Journal of Rehabilitation Medicine. 2013;28(9):830-833.

[16] Liu JY. Clinical study on the effects of core muscle group training on balance disorders in stroke patients . Chengdu: Chengdu University of Traditional Chinese Medicine; 2014:20-45.

[17] Zeng ZB, Qin SF, Zhan SJ. Effects of core strength training on motor function and balance in stroke patients [In Chinese]. Modern Diagnosis & Treatment. 2016;27(19):3705-3707.

[18] Cabanas-Valdés R, Bagur-Calafat C, Girabent-Farrés M, et al. The effect of additional core stability exercises on improving dynamic sitting balance and trunk control for subacute stroke patients: A randomized controlled trial. Clinical Rehabilitation. 2016;30(10):1024-1033.

[19] He YG, Zhang T. Effects of intensive sit-to-stand training on balance and walking ability in post-stroke hemiplegia patients. Chinese Journal of Physical Medicine and Rehabilitation. 2012;34(8):596-599.

[20] Chu KS, Eng JJ, Dawson AS, et al. Water-based exercise for cardiovascular fitness in people with chronic stroke: A randomized controlled trial. Arch Phys Med Rehabil. 2004;85(6):870-874.

[21] Ku PH, Chen SF, Yang YR, et al. The effects of Ai Chi for balance in individuals with chronic stroke: A randomized controlled trial. Sci Rep. 2020;10(1):1201.

[22] Tripp F, Krakow K. Effects of an aquatic therapy approach on functional mobility in subacute stroke patients: A randomized controlled trial. Clinical Rehabilitation. 2014;28(5):432-439.

[23] Zhu ZZ, Cui LL, Yin MM, et al. Hydrotherapy vs. conventional land-based exercise for improving walking and balance after stroke: A randomized controlled trial. Clinical Rehabilitation. 2016;30(6):587-593.

[24] Lee SY, Im SH, Kim BR, et al. The effects of a motorized aquatic treadmill exercise program on muscle strength, cardiorespiratory fitness, and clinical function in subacute stroke patients: A randomized controlled pilot trial. Am J Phys Med Rehabil. 2018;97(8):533-540.

[25] Eyvaz N, Dundar U, Yesil H. Effects of water-based and land-based exercises on walking and balance functions of patients with hemiplegia. NeuroRehabilitation. 2018;43(2):237-246.

[26] Zeng M, Cui Y, Wang YL, et al. Effects of underwater treadmill walking training on walking function in stroke patients. Chinese Journal of Rehabilitation Theory and Practice. 2019;25(1):76-80.

[27] Wang L, Dai CQ. Effects of intensive water-based walking training on recovery of walking ability in post-stroke hemiplegia patients. Chinese Journal of Rehabilitation Medicine. 2014;29(1):76-78.

[28] Xie BJ, Yang M, Bai YL. Clinical study of Baduanjin on motor function recovery in stroke patients . West China Medicine. 2019;34(5):515-519.

[29] Fu CX, Zhang QY. Effects of Tai Chi on balance function and walking ability in post-stroke hemiplegia patients. Chinese Journal of Rehabilitation Medicine. 2016;31(5):536-539.

[30] Liu TJ, Qin P, Chen XZ. Observation of Tai Chi in improving balance function in stroke patients . Chinese Journal of Physical Medicine and Rehabilitation. 2009;31(11):781-782.

[31] Wang XB, Hou MJ, Tao J, et al. Correlation study of Tai Chi “Cloud Hands” and gait in post-stroke hemiplegia patients. Chinese Journal of Rehabilitation Medicine. 2016;31(12):1328-1333.

[32] Yang HX, Tang Q. Clinical observation of Tai Chi for rehabilitation of motor dysfunction after stroke. Chinese Journal of Rehabilitation Medicine. 2016;31(10):1146-1148.

[33] He J, Wang W, Li KP, et al. Effects of six-form Tai Chi training on postural balance function in stroke patients. Chinese Journal of Rehabilitation Medicine. 2022;37(4):482-487.

[34] Büyükavcı R, Şahin F, Sağ S, Doğu B, Kuran B. The impact of additional trunk balance exercises on balance, functional condition and ambulation in early stroke patients: Randomized controlled trial. Turk J Phys Med Rehabil. 2016;62(3):248-256.

[35] Ordahan B, Karahan AY, Basaran A, Turkoglu G, Kucuksarac S, Cubukcu M, Tekin L, Polat AD, Kuran B. Impact of exercises administered to stroke patients with balance trainer on rehabilitation results: A randomized controlled study. Hippokratia. 2015;19(2):125-130.

[36] Yoo SD, Jeong YS, Kim DH, Lee MA, Noh SG, Shin YW, Kim HS, Kim SH. The efficacy of core strengthening on the trunk balance in patients with subacute stroke. Ann Rehabil Med. 2010;34(6):677-682.

[37] Globas C, Becker C, Cerny J, et al. Chronic stroke survivors benefit from high-intensity aerobic treadmill exercise: A randomized control trial. Neurorehabilitation and Neural Repair. 2012;26(1):85-95.

[38]Lamberti N, Straudi S, Malagoni AM, et al. Effects of low-intensity endurance and resistance training on mobility in chronic stroke survivors: a pilot randomized controlled study. Eur J Phys Rehabil Med. 2017;53(2):228-239.

[39] Lau KWK, Mak MK. Speed-dependent treadmill training is effective to improve gait and balance performance in patients with sub-acute stroke. J Rehabil Med. 2011;43(8):709-713.

[40] Moore JL, Nordvik JE, Erichsen A, et al. Implementation of high-intensity stepping training during inpatient stroke rehabilitation improves functional outcomes. Stroke. 2020;51(2):563-570.

[41] Pang MY, Eng JJ, Dawson AS, et al. A community-based fitness and mobility exercise program for older adults with chronic stroke: A randomized, controlled trial. J Am Geriatr Soc. 2005;53(10):1667-1674.

[42]Gjellesvik TI, Becker F, Tjønna AE, et al. Effects of High-Intensity Interval Training After Stroke on Physical and Cognitive Function: A Multicenter Randomized Controlled Trial. Arch Phys Med Rehabil. 2021;102(9):1683-1691. doi:10.1016/j.apmr.2021.05.008
